# Identification of Sponge-Associated Bacteria From the Coast of Kuwait and Their Potential Biotechnological Applications

**DOI:** 10.3389/fmicb.2022.896718

**Published:** 2022-07-04

**Authors:** Shahad Abbas, Huda Mahmoud

**Affiliations:** Department of Biological Sciences, Faculty of Science, Kuwait University, Safat, Kuwait

**Keywords:** Arabian Gulf, sponge-associated bacteria, biotechnological potentials, microbial diversity, biomineralization

## Abstract

Sponges are among the most ancient animals harboring complex microbial communities with potential applications in biotechnology. The Arabian Gulf is a thermally stressed enclosed body of water located in an arid region where sponges and their halobionts are understudied. This study combined 16S rRNA next-generation gene amplicon sequencing and cultivation techniques to explore the abundance and diversity of sponge-associated bacteria. Culture-independent techniques showed the associations of more than 25 bacterial phyla with *Amphimedon* sp., *Chondrilla australiensis, Haliclona* sp., and *Niphates* spp. Regarding cultivable bacteria, 315 bacterial isolates associated with the sponge *Haliclona* sp. were cultivated; these isolates were affiliated with the phyla Proteobacteria and Firmicutes and were distributed among six bacterial genera. Selected strains of *Bacillus*, *Ferrimonas*, *Pseudovibrio*, *Shewanella*, *Spongiobacter,* and *Vibrio* were tested for antimicrobial activity against indicator microorganisms and protease enzyme production. Seven *Bacillus* strains exhibited weak to moderate growth inhibition against *Bacillus subtilis, Staphylococcus aureus,* and *Candida albicans*. Furthermore, 29 different strains of *Bacillus, Ferrimonas*, *Shewanella*, and *Vibrio* exhibited different degrees of positive protease activity. In addition, cultivated strains of *Bacillus*, *Shewanella*, *Pseudovibrio,* and *Vibrio* were tested for their biomineralization abilities. Herein we report for the first time the isolation of biomineralizing bacteria from sponge tissue where eleven bacterial isolates produced different shapes of calcium carbonate crystals on agar. Our observations shed light on the diversity and biotechnological potentials of sponges-associated bacteria inhabiting one of the world’s hottest seas.

## Introduction

Marine sponges (Phylum Porifera) are one of the oldest groups of multicellular organisms, having originated in the Precambrian era (Li et al., 1998; Van Soest et al., 2012). They are distributed worldwide and occupy various surfaces in both shallow- and deep-water regions and play several functional and ecological roles in coral reef systems; for example, they promote coral survival by binding them to the reef frame and preventing excavating organisms from entering and destroying the reefs skeletons (Wulff, 2001). Some sponges are involved in nutrient recycling in the reef through being mixotrophic, which refers to the sponge ability to feed heterotrophically on bacterioplankton and phytoplankton while their photosynthetic endosymbionts provide them with the required nutrients to survive. Therefore, sponges can provide a food source for other organisms in the marine environment (Wulff, 2001). In one of the most ancient known microbial-metazoan symbioses, sponges establish close, complex associations with diverse microbial consortia, including bacteria (Lee et al., 2011; Giles et al., 2013), archaea (Sharp et al., 2007; Turon and Uriz, 2020), and fungi (Maldonado et al., 2005; Proksch et al., 2008; Nguyen and Thomas, 2018). Sponge-associated microorganisms can comprise approximately 40% of the host biomass and show various functions, such as photosynthesis (Wilkinson, 1983), nitrogen fixation (Wilkinson and Fay, 1979), sulfate reduction (Hoffmann et al., 2005) and secondary active metabolite production (Graça et al., 2015). Sponge-associated bacteria are expected to produce antimicrobial substances to compete and to maintain stable population (Esteves et al., 2017) while the sessile sponges depend on the secondary metabolites as a defensive mechanism against potential threats (Becerro et al., 1994; Chanas et al., 1997; Engel and Pawlik, 2000). Thus, these animals represent a hidden treasure trove of novel metabolites with biotechnological potential (Bull and Stach, 2007). Over the years, more than 5,000 compounds have been isolated from about 500 sponge species and continuing (Müller et al., 2004; Han et al., 2019; Varijakzhan et al., 2021); as a result, interest in investigating and cultivating sponge-associated microbiomes has increased over the years, as sponge-associated microbiomes are probably the true source of these natural products (Proksch et al., 2002; Taylor et al., 2007).

Researchers have investigated sponge-associated microorganisms by combining culture-dependent and culture-independent techniques (Sun et al., 2010; Jackson et al., 2012; Kuo et al., 2019). To increase the diversity of cultivable bacteria, several studies on different sponge species from different locations have used various pretreatment techniques and different culture media. These studies succeeded in the cultivation of bacteria affiliated with the phyla Proteobacteria, Actinobacteria, Firmicutes, Bacteroidetes, Planctomycetes and Verrucomicrobia (see Supplementary Table S1). However, due to limitations associated with culture-dependent techniques (Sun et al., 2010) where they are biased against the majority of unculturable microbes that can reach to 99% of any environment (Staley and Konopka, 1985), culture-independent techniques provide more insight into the diversity of sponge bacterial communities. Molecular techniques revealed the association of more than 60 bacterial and archaeal phyla associated with 268 different sponge species, where each sponge sample hosted at least a total of 13 different phyla (Moitinho-Silva et al., 2017). Studies using polymerase chain reaction along with denaturing gradient gel electrophoresis (PCR-DGGE), cloning and sequencing of the 16S rRNA gene fragments proved that different sponge species were dominated by various bacterial phyla, including Acidobacteria, Actinobacteria, Bacteroidetes, Chloroflexi, Cyanobacteria, Gemmatimonadetes, Nitrospirae, and Proteobacteria (α, β, δ, and γ; Schmitt et al., 2007; Weisz et al., 2007; Yang et al., 2011; Simister et al., 2012; Kuo et al., 2019; Moreno-Pino et al., 2020), some of which are considered microbial signatures commonly associated with sponges.

Studies are available on sponge-associated bacteria from arctic, temperate, tropical, and subtropical regions (Simister et al., 2012; Luter et al., 2015; Villegas-Plazas et al., 2019; Moreno-Pino et al., 2020), while research on sponges from the Arabian Gulf is scarce, and the only available data using next generation sequencing techniques come from Najafi et al. (2018). The Arabian Gulf is a semi enclosed body of water located in an arid region of the subtropical zone that is characterized by a shallow basin with an average depth of approximately 35 m. The Arabian Gulf is characterized by high salinity and extreme temperature fluctuations, especially in the northwestern region (Sheppard, 2009).

As sponge studies are limited in the northwestern region of the Gulf, particularly in Kuwait, our research combined culture-dependent and culture-independent techniques to investigate the diversity of sponge-associated bacteria collected from the Kuwait marine environment and to explore the potential biotechnological abilities of cultivable bacteria from sponges. Due to the extreme nature of the Gulf, the sponge inhabiting the region may harbor unique microbes with unique functions reflecting their metabolic specialization.

## Materials and Methods

### Sponge Sampling and Treatment

Sponge samples were collected from offshore and sub-intertidal area in Kuwait marine environment. Five sponge samples were collected from Kubbar Island (N 29°02′53.6 E 48°17′23.3), and two samples were collected from Nuwaiseeb (N 28°32′38.8 E 48°25′22.2) sub-intertidal area. Fragments of sponge individuals from Kubbar Island were collected by SCUBA diving at a depth of 9 m, while fragments of sponge individuals from Nuwaiseeb sub-intertidal area were collected by hand. Sponge fragments used for microbiomes investigation were transferred to a sterile 50 ml tubes containing 95% ethanol. On the other hand, samples used for cultivating sponge-associated bacteria were kept in seawater in sterile 50 ml tubes. All samples were kept in ice, shipped to the laboratory, and processed immediately.

### Sponge Identification

Sponge taxonomic identification was done in collaboration with sponge taxonomist Dr. Nicole de Voogd from the Naturalis Biodiversity Center in Leiden, Netherlands (Abbas, De Voogd and Mahmoud, in preparation). The examination and identification were carried out in Bio partner laboratory in the University of Leiden where samples were identified based on their distinct macroscopic morphology and spicules characters. Sponges’ pictures were obtained on sampling site and the preparation of sponge spicules using digestion method and histological sections of sponge skeleton were made and observed under light and scanning electron microscopy. Morphological characters of sponge spicules type, shape, and size were documented along with the structural arrangements of sponge skeleton (Boury-Ensnault and Rützler, 1997; Hooper and Van Soest, 2002). The characters of our collection were compared and crosschecked with previously Identified sponge species from World Porifera Database (WPD; Van Soest et al., 2018) and “Systema Porifera” a guidebook to the classification of sponges (Hooper and Van Soest, 2002).

### Total Genomic DNA Extraction and 16S rRNA Next Generation Sequencing

Total Genomic DNA of seven sponge samples were extracted using DNeasy Power Soil Kit (Qiagen, Germany). Sponge samples were rinsed multiple times using sterile 0.2 μm Millipore filtered seawater. Approximately 4 g of each sponge sample were cut and macerated separately using a sterile mortar and pestle. Sponge macerate was transferred to bead beating tubes with 200ul of 50 mg ml^−1^ lysozyme (Sigma, Germany). The tubes were incubated in a water bath at 37°C for 30 min. Afterward, 20 μl of proteinase K (Sigma, USA) was added and the tubes were incubated in a water bath at 55°C for 10 min. The rest of the extraction steps were done following the manufacturer protocol. The DNA quality was determined using nanodrop spectrophotometry and Qubit fluorimeter.

The 16S rRNA next generation sequencing (Illumina-MiSeq) of the extracted DNA was sent to SEQme Company facility (Dobris, Czech Republic). According to manufacturer’s instructions, The V3-V4 region of domain bacteria 16S rRNA amplicon library was generated using the NEBNext^®^ Ultra^™^ II DNA Library Prep Kit for Illumina (New England Biolabs, USA) and NEBNEX multiplex oligos for Illumina. The V3-V4 region of the 16S rRNA was amplified using the primers V3-V4F357 (3’CCTACGGGNGGCWGCAG’5) and V3-V4R805 (3’GACTACHVGGGTATCTAATCC’5). Agilent High Sensitivity DNA Kit on Bioanalyzer 2100 was used and the final library quality control was performed and quantified by qPCR using the KAPA Library Quantification Kit (Kapa Biosystems, USA). Illumina MiSeq (Illumina Hayward, USA) was used for sequencing for 251 cycles by a MiSeq Reagent Kit V2 (Illumina, USA) and PhixControl Kit V3 (Illumina, USA). Raw data were processed by BaseSpace cloud interface (Illumina). Barcode sequences of raw reads were trimmed by FASTX-toolkit (v 0.0.14) and each sample (R1 and R2) reads were merged to one file using fastq-join (v 1.1.2) and then converted to fasta format (FASTX Toolkit v 0.0.14). The same number of reads (i.e., 32.500 reads) were analyzed for each sponge sample to remove biases and compare richness among samples. The data set were processed by using NGS analysis pipeline of the SILVA rRNA gene database project (SILVAngs 1.3; Quast et al., 2013) and the primer sequence was removed during SILVAngs analysis pipeline. Reads were aligned individually using the SILVA Incremental Aligner (SINA; SINA v1.2.10 for ARB SVN revision 21008; Pruesse et al., 2012) against the SILVA SSU rRNA SEED and were quality controlled (Quast et al., 2013). Reads shorter than 50 aligned nucleotides, or have more than 2% of ambiguities (i.e., The maximum relative number of ambiguous bases that is allowed before a sequence were rejected), or 2% of homopolymers, or had putative contaminations, or artifacts reads with a low alignment quality (50 alignment identity, 40 alignment score reported by SINA) were excluded from further processing. Using VSEARCH (version 2.14.2^1^; Rognes et al., 2016), identical reads were identified (dereplication), and unique reads were clustered (Operational taxonomic units OTUs). BLASTn (version 2.2.30+)^2^ was used and the reference read of each OTU was classified by a local nucleotide BLAST search against the non-redundant version of the SILVA SSU Ref dataset (release 128^3^; Camacho et al., 2009). Quantitative information (number of individual reads per taxonomic path) was produced as the classification of each OTU reference read was mapped onto all reads that were allocated to the respective OUT. Reads were assigned to the meta group “No Relative” in the SILVAngs fingerprint because they were without any or weak classifications, where the function “(% sequence identity + % alignment coverage)/2″ did not exceed the value of 93 (Ondov et al., 2011). This method was first used in the publications of Klindworth et al. (2013) and Ionescu et al. (2012). Upon the processing of the raw data, reads were clustered into operational taxonomic units (OTUs). The sequences showed variation in the total number of OTUs and bacterial genera in the studied sponges. Furthermore, the data revealed the presence of one or two individuals/sequences of certain microorganisms in the samples, referred to as singletons or doubletons, which were eliminated before processing the data.

The amplicon metagenome data were submitted in the GenBank under the accession number PRJNA540061.

### Enumeration of Culturable Bacterial Associated With *Haliclona* sp. 1Kl

Two grams of pre-washed *Haliclona* sp. (1KI) sponge tissue was macerated under aseptic conditions using a sterile mortar and pestle. Sponge macerate was diluted up to 10^−5^ using sterile saline (i.e., 3% NaCl) followed by spreading an aliquot of 0.1 ml of each dilution on marine agar (MA; HIMEDIA, India). The test was done in triplicates and plates were incubated at 30°C for 24 h. The colonies were counted, sub-cultured and purified using MA where the morphological and cultural characteristics of the purified bacterial colonies were documented and used for screening and selecting colonies for further testing (Supplementary Table S2). Culture and morphological (microscopic) characters of isolates were compared and used to categorise the isolates into different groups and represents from each group were chosen for sequencing.

### DNA Extraction and Sequencing of Bacterial Isolates

Total genomic DNA from selected pure bacterial cultures were extracted using Prepman^™^ Ultra Kit (Applied Biosystems, United States) following the manufactures protocol. The extracted DNA from purified bacterial culture was amplified using Polymerase chain reaction (PCR). Using Ready-To-Go PCR beads (GE Healthcare, UK), the partial 16S rRNA was amplified using universal bacterial primers. Each tube contained 25 ng of extracted DNA, 30 pmole of each of the reverse 907R (5^’^-CCGTCAATTCMTTTGAGTTT-3^′^) and the forward 341F (5^’^-CCTACGGGAGGCAGCAG-3^′^) universal bacterial primers (Sigma, Germany; Muyzer et al., 1993) and 23.5 μl of molecular water. PCR amplification was performed in a Thermocycler GeneAmp PCR system 9,700 (Applied Biosystems, United States). The amplification program started with an initial denaturation step at 94°C for 30 s followed by annealing at 55°C for 30 s, an extension step at 72°C for 30 s, and a final extension step at 72°C for 7 min. The PCR product was purified using QIAquick PCR purification Kit (Qiagen, United States) following the manufacturer protocol and the DNA was labelled using BigDye 3.1 V Kit (Applied Biosystems, United States). One microliter f 341F primer or 907R primer, 2 μl purified PCR product, 2 μl of 5x sequencing buffer, 2 μl BigDye 3.1 V reagent and 3 μl molecular water were mixed until the total volume of the mixture reached 10 μl. Incubated in the thermocycler (Applied Biosystems, USA), the program consisted of 25 cycles of denaturation at 96°C for 10 s, annealing at 55°C for 5 s, and extension at 60°C for 4 min. The product was purified using 2 μl of 3 M Na-acetate (pH 5.2) and 50 μl of absolute ethanol was added to the tubes and kept in the dark for 20 min. The tubes were centrifuged for 15 min at maximum speed and the pellet was washed with 50 μl of 70% ethanol, centrifugation for 5 min at high speed, supernatant discarded and finally the pellet was air dried for 15 min. The product was analyzed using a 31301*xl* genetic analyzer (Applied Biosystems, United States) and the sequences were collected and compared in the GenBank using nucleotide BLAST. The sequences were deposited in the GenBank under the accession number (MK558635-MK558695; Supplementary Table S3).

### Screening of *Haliclona* sp. Cultivated Bacterial Isolates in the Amplicon Data

The sequences of the bacterial isolates obtained from the *Haliclona* sp. 1KI sponge tissue were screened against the same sponge amplicon data deposited in the NCBI GenBank (Accession number SRX14840950). The match was done using the sequence read archive (SRA) search engine facility, and the number of times the bacterial isolates sequences were detected in the amplicon data was documented.

### Antimicrobial Assay

Agar-well-diffusion method (Isaacson and Kirschbaum, 1986) was used to investigate the antimicrobial activity of selected *Haliclona* sp. cultivated bacterial isolates. The test was performed against a group of Gram-positive bacteria (i.e., *Staphylococcus aureus, Bacillus subtilis*), Gram-negative bacteria (i.e., *Escherichia coli, Pseudomonas aeruginosa*) and yeast (i.e., *Candida albicans, Saccharomyces cerevisiae*). An aliquot of 0.1 ml of Gram-positive, Gram-negative bacteria were spread on Nutrient Agar (NA; Difco) and yeast on Potato Dextrose Agar (PDA; Difco) media. Three holes were made in the agar and filled with 10 μl filtrate of cultivated bacterial isolates grown in Marine Broth (MB; Difco) and harvested at their mid lag phase. The test was repeated in triplicate. The filtrate was prepared by filtrating the inoculated MB using sterile 0.2 μm membrane filter (Millipore). The NA plates were incubated at 37°C for 24 h and PDA plates at 30°C for 24 h. Both negative control (uninoculated MB) and 5 mg ml^−1^ positive control (commercially available antimicrobial drugs Ampicillin, Kanamycin, Penicillin-G and Cycloheximide, Sigma) were included in the assessment. The inhibition zones formed around the wells were measured and documented.

### Protease Activity

Milk agar (HIMEDIA, India) modified with 3% NaCl was used to investigate the protease activity of cultivated sponge-associated bacteria. Selected bacterial isolates were streaked on Milk Agar and incubated at 30°C for 24 h. After the incubation period, the plates were flooded with Coomassie blue stain and incubated at room temperature for 20 min followed by a gentle wash with distilled water and then de-stained with acetic acid (Mazotto et al., 2013). The inhibition zone surrounding the plates were observed and measured.

### Biomineralization Ability

Modified B4 medium (4 g yeast extract, 2.5 g calcium acetate, and 15 g agar; Boquet et al., 1973) was used to observe mineral precipitation by bacterial isolates. Grown in 3% NaCl marine broth, an aliquot of 0.1 ml of selected pure bacterial cultures was spread on B4 medium. The plates were sealed with parafilm and incubated at 30°C for two months. For crystal harvesting, plates were flooded with absolute ethanol for 5 min. Small square block of inoculated B4 media were cut and added to 15 ml tube filled with sterile distilled water. The tube was then incubated in water bath at 100°C until the agar melted and then centrifuged (Eppendorf, United States) at 14,000 rpm in room temperature to prevent the agar from solidifying. The water was discarded, and 0.5 ml of sodium hypochlorite (bleach) was added, and the crystal/bleach mixture was mixed gently. The mixture was transferred to Eppendorf tube and centrifuged at maximum speed for 2 min. Bleach was pipetted out and distilled water was added instead and centrifuged for 2 min at 13,200 rpm speed. The cleaning steps was repeated twice followed by pipetting out the water and adding 70% ethanol (Mahmoud, 2015). Crystals were prepared for scanning electron microscope (SEM) where few drops of ethanol/crystals mixture were added to a double-sided tape on SEM stubs. Left to air dry, the stubs were mounted with a thin 5 nm layer of gold using LEICA EM ACE 200 sputter coating machine (LEICA, Germany) and the crystals were observed using JOEL scanning electron microscope (JEOL, United States).

## Results

### Sponge Identification

A total of seven sponge samples were collected from two different locations in Kuwait. Six sponge samples were identified to the genus level, and one sample was identified to the species level under the supervision of the sponge taxonomist Dr. Nicole de Voogd. The taxonomic identification classified all sponge samples under the class Demospongiae and order Haplosclerida, and they and belonged to the families Niphatidae, Chalinidae, and Chondrillidae. Three samples were classified as novel species of the genus *Niphates*, designated sp. 1 and sp. 3; two novel samples of the genus *Haliclona* and one novel species of the genus *Amphemideon* (Supplementary Figure S1; Supplementary Table S4).

### Sponge-Associated Bacteria

The raw sequencing of bacterial 16S rRNA gene fragments from five Kubbar Island sponges and two Nuwaiseeb sponges generated a total of 1,580,395 reads. The greatest number of reads was obtained from *Niphates* sp. 3 2KI, which produced 402,525 reads, followed by *Haliclona* sp. 1KI, with 316,199 reads. In contrast, *Amphimedon* sp. 1 6KI produced a total of 74,801 reads, which was the lowest number of reads among all investigated sponge samples. Furthermore, the number of species of the sponge prokaryotic communities was assessed by constructing a rarefaction plot. As shown in Supplementary Figure S2, the rarefaction curves of the tested sponge samples plateaued, except for that of *Amphimedon* sp. 6KI, indicating that sequencing reached complete saturation (all microbiome DNA were well sampled and sequenced) in 6 out of the 7 investigated sponge samples.

In this study, the total number of OTUs of the tested sponge samples ranged between (1,147-3,708). In addition to the total number of OTUs, the total numbers of bacterial genera associated with the tested sponge samples ranged between (119–440). The number of reads, OTUs and genera from each sponge sample are presented in Supplementary Table S5.

**Figure 1:**
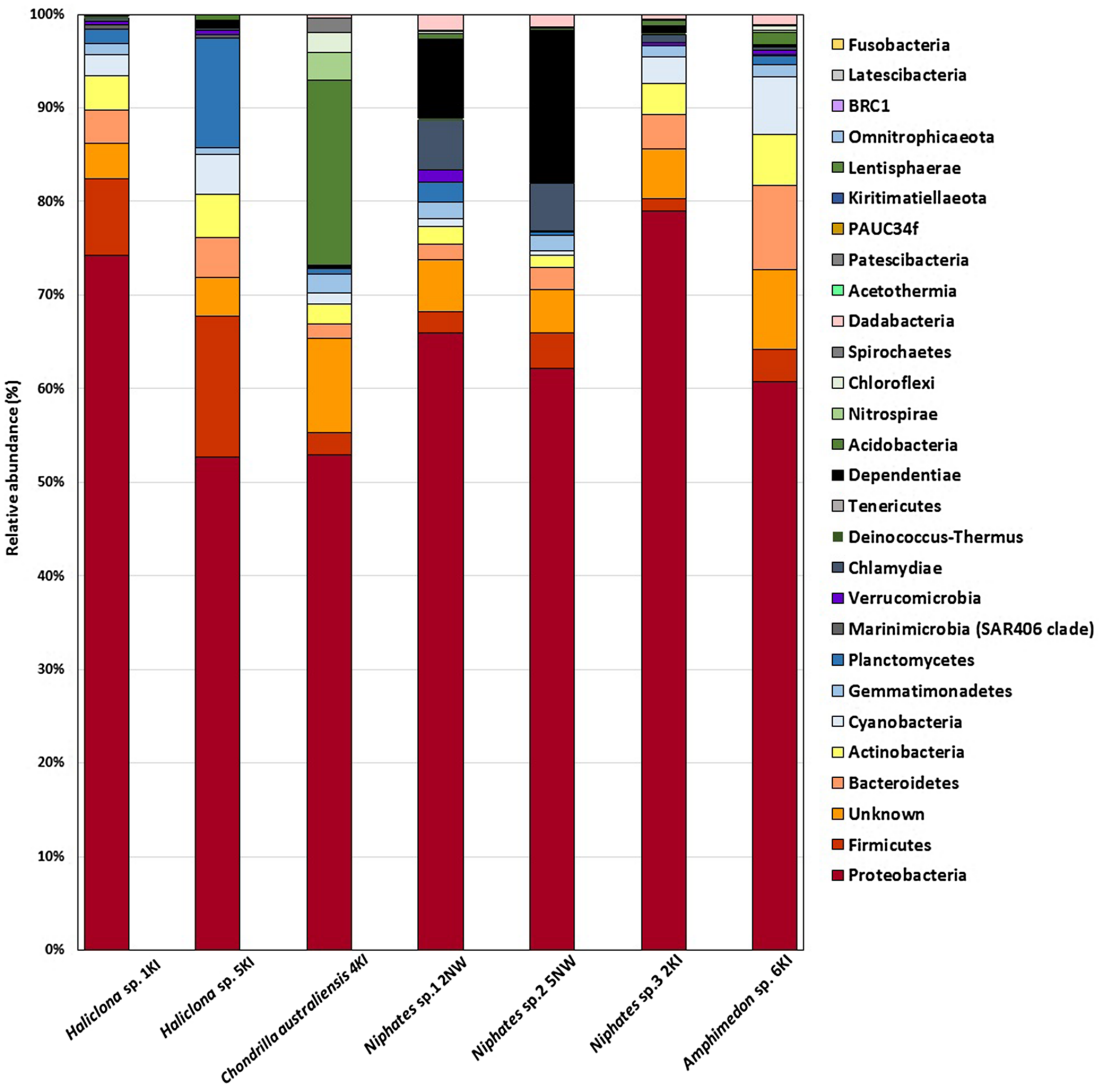
Bar charts represent the bacterial phyla associated with sponge samples in the present study.

A total of 13,778 OTUs from 7 sponge samples were distributed among more than 25 bacterial phyla. In the current study, most sponge-associated bacteria were affiliated with the phyla Proteobacteria, Bacteroidetes, Firmicutes, Dependentiae, Actinobacteria, Acidobacteria, Cyanobacteria, Planctomycetes, Chlamydiae, Chloroflexi, Verrucomicrobia, Nitrospirae, and Gemmatimonadetes. As shown in Figure 1, the sponge samples shared at least 4 bacterial phyla, among which Proteobacteria was associated with all sponge samples, with percentages ranging between 53 and 79%. The remaining bacterial phyla associated with the tested sponges contributed with certain sponge species at lower percentages. Along with Proteobacteria, these bacterial phyla are considered major phyla and can be seen associated with almost all of our tested sponge samples such as Firmicutes, Bacteroidetes, Actinobacteria, Acidobacteria, Cyanobacteria, Planctomycetes, Chloroflexi, Dependentiae and Chlamydiae. In addition to the most dominant bacterial phyla, 4–10% of the OTUs in various sponge samples were belonged to unknown (unassigned) groups, as shown in the bar charts. In addition to the most dominant bacterial phyla, a total of 14 minor bacterial phyla and candidate phyla with less than 1% relative abundance were found to be associated with the tested sponge samples. These OTUs belonged to the phyla Marinimicrobia, Deinococcus-Thermus, Tenericutes, Patescibacteria, Dadabacteria, BRC1, Spirochaetes, Fusobacteria, Latescibacteria, Kiritimatiellaeota, Lentisphaerae, Omnitrophicaeota, Acetothermia and PAUC 34f (Figure 1).

**Figure 2:**
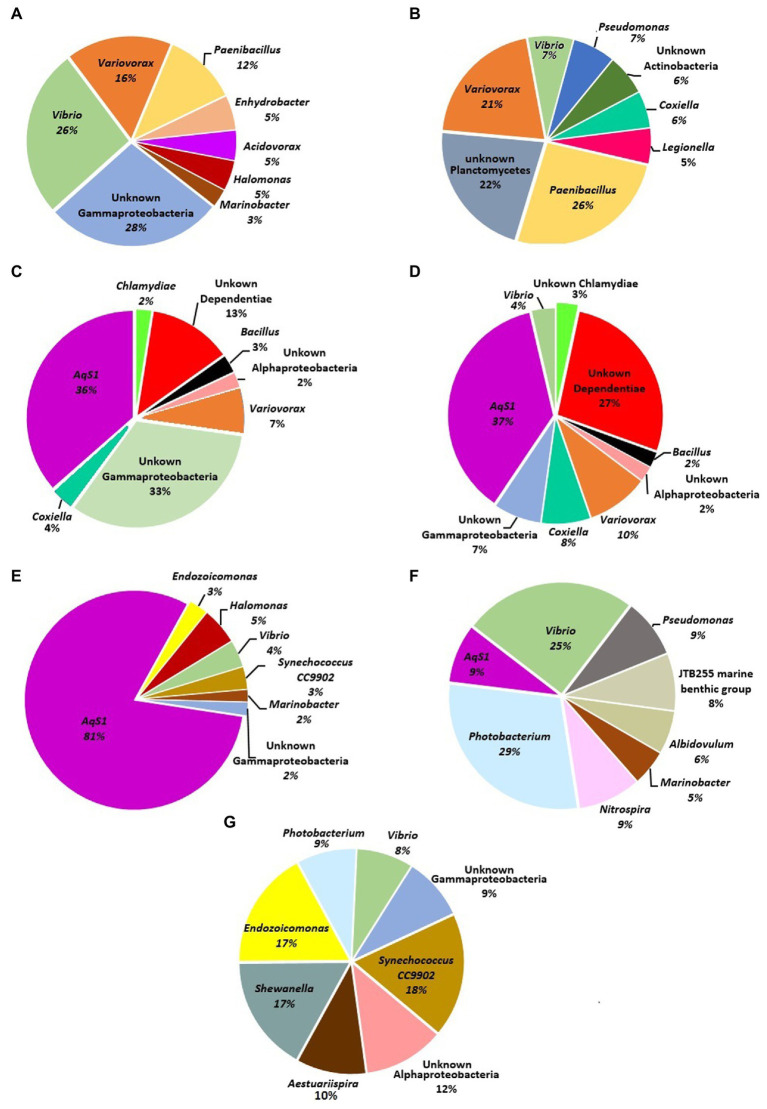
Pie charts represent most dominant bacterial genera associated with sponge samples in the present study. **(A)** *Haliclona* sp. 1KI, **(B)** *Haliclona* sp. 5KI **(C)** *Niphates* sp.1 2NW, **(D)** *Niphates* sp.2 5NW, **(E)** *Niphates* sp.3 2KI, **(F)** *Chondrilla australiensis* 4KI, and **(G)** *Amphimedon* sp. 6KI.

The most dominant bacteria associated with each sponge sample were also determined at the genus level (Figure 2). Various bacterial genera such as; *Variovorax*, *Paenibacillus, Vibrio*, *Coxiella*, *Bacillus, Synechococcus* CC9902, *Shewanella*, *Endozoicomonas*, *Photobacterium*, *Aestuariispira, Photobacterium*, *Nitrospira*, *Pseudomonas*, *Albidovulum*, *Marinobacter* and the novel chromatials genus AqS1 were some of the most dominant genera in the test. In addition to the previously mentioned bacterial genera, a large number of the bacterial OTUs detected in different sponge samples belonged to unknown genera of Alpha and Gammaproteobacteria, Actinobacteria, Dependentiae, Chlamydia and Planctomycetes.

### Detecting Cultivated Bacterial Isolates Sequences in *Haliclona* sp. 1Kl Amplicon Bacteriome Data

Bacterial isolates sequences were screened against *Haliclona* sp. 1Kl bacteriome amplicon data. The aim was to ensure that the origin of the isolates was indeed the sponge tissue and not the adjacent seawater. Supplementary Table S6 shows the detection of all isolates in the amplicon. Some *Ferrimonas* sp. and *Vibrio* sp. isolates showed many matches in the amplicon data, reaching 1728 and 1,371, respectively. On the other hand, some bacterial isolates sequences were detected only once in the amplicon data, such as the case with *Pseudovibrio* sp.*, Spongiobacter* sp., and *Bacillus* sp. (Supplementary Table S6).

### Biotechnological Potential of *Haliclona* sp. 1KI-Associated Culturable Bacteria

The number of colony forming units (CFUs) isolated from *Haliclona* sp. 1KI sponge tissue was 2 × 10^4^ ± 0.2 CFU g^−1^ [mean (μ) ± standard error (SE)].

**Figure 3:**
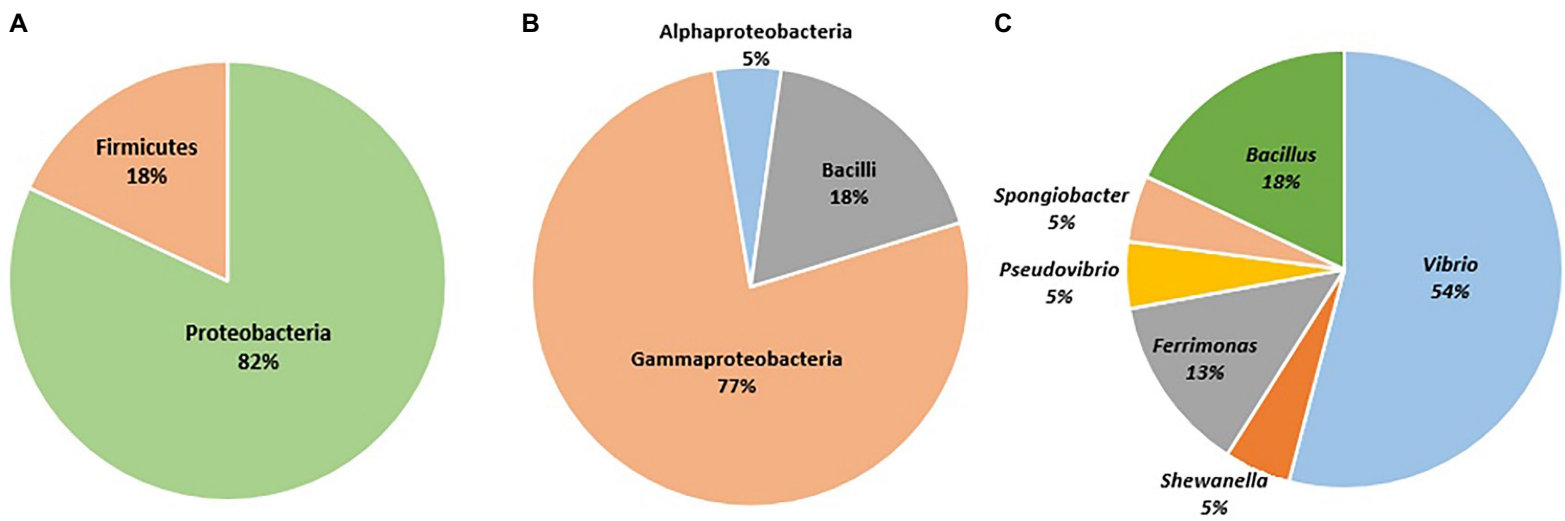
Sequenced bacterial isolates identified to the **(A)** phylum level, **(B)** class level, and **(C)** genera level.

A total of 315 bacterial isolates were obtained. Based on the variation in their culture characteristics and morphological features, a total of 61 bacterial isolates were chosen for sequencing. The sequenced isolates showed more than 97% similarity to their closest relatives in GenBank. A total of 82% of the sequenced isolates were assigned to the phylum Proteobacteria, while the remaining 18% of the sequenced isolates were affiliated with the phylum Firmicutes. The sequenced isolates were assigned to 3 classes and 6 genera. Among the 6 genera (Figure 3), *Vibrio* sp. accounted for the highest number of isolates (*n* = 33 out of 61), followed by species of *Bacillus*, *Ferrimonas*, *Shewanella*, *Pseudovibrio*, and *Spongiobacter*. The bacterial strains isolated from *Haliclona* sp. 1KI provided evidence of its abilities that may be utilized in various biotechnological applications. Furthermore, several matches were found upon comparing the culturable bacterial sequences with the *Haliclona* sp. 1Kl amplicon data (Supplementary Table S6); hence the potential source for cultivating these isolates is the sponge tissue and not the seawater.

### Antimicrobial Activity of Sponge-Associated Bacteria

**Figure 4:**
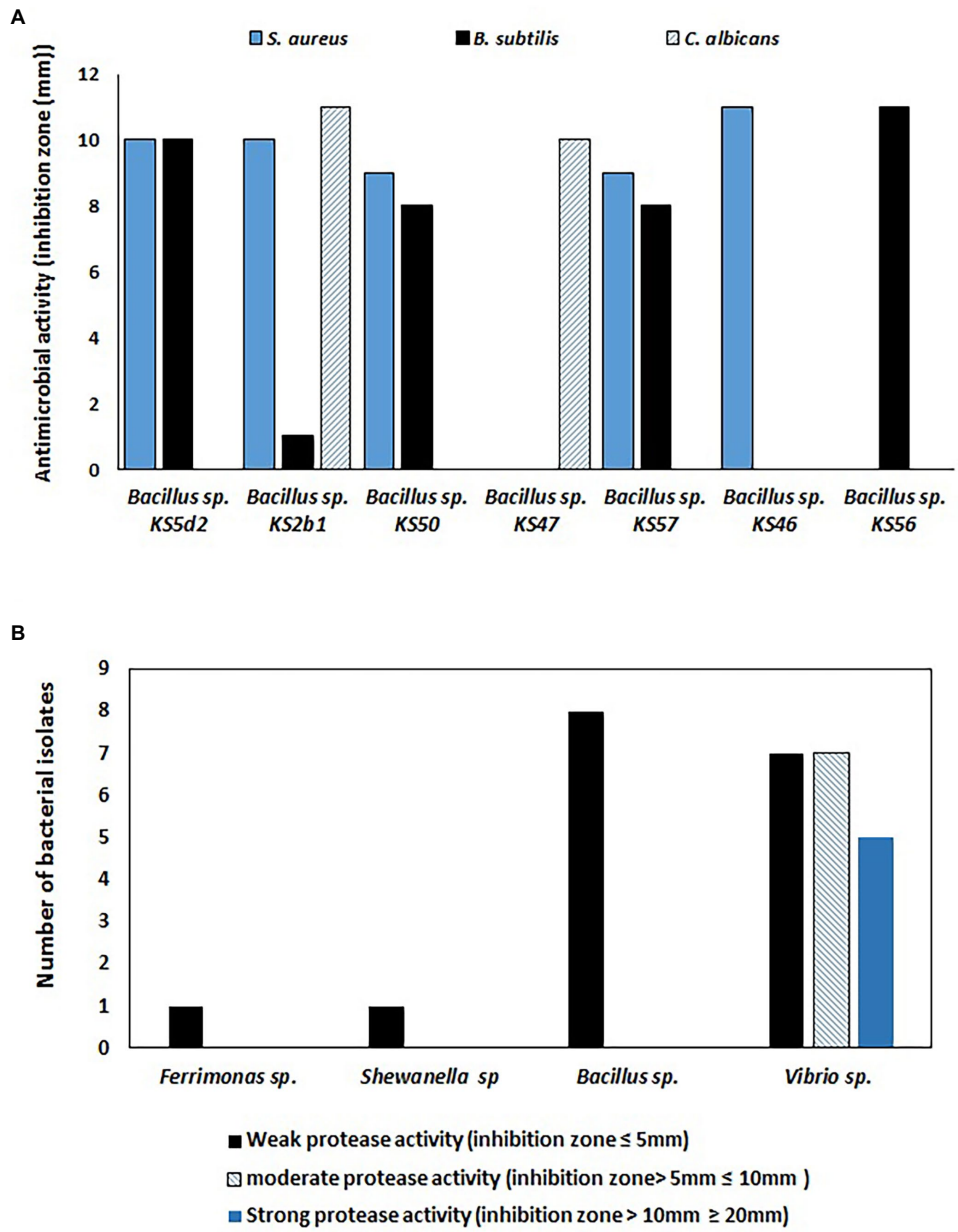
**(A)** Antimicrobial activities of sponge-associated bacterial isolates against three bacterial cultures (i.e., *Bacillus subtilis*, *Staphylococcus aureus*, and *Candida albicans*). Weak antimicrobial activity (inhibition zone ≤10 mm) and moderate antimicrobial activity (inhibition zone ≥10 mm), **(B)** Number of bacterial isolates with positive protease activity.

In the current study, a total of 53 selected bacterial isolates were tested for antimicrobial activity, and 13% (*n* = 7) of isolates showed positive activity against at least one tested culture (i.e., *S. aureus, B. subtilis*, *E. coli, P. aeruginosa, C. albicans*, and *S. cerevisiae*). As shown in Figure 4A and Supplementary Table S7, seven *Bacillus* sp. isolates with positive antimicrobial activity showed two ranges of inhibition zones indicating weak to moderate activity against *S. aureus*, *B subtilis* and *C. albicans* only. Among the seven *Bacillus* sp. isolates with positive antimicrobial activity, three isolates showed weak or moderate activity against *S. aureus* and *B. subtilis,* while three isolates showed moderate activity against one tested strain. On the other hand, only one *Bacillus* sp. isolate showed positive activity against all tested cultures.

### Production of Protease Enzyme

Among 50 tested bacterial isolates, 29 bacteria exhibited positive protease activity. The bacterial isolates with positive protease activity produced a clear zone, which was considered to indicate weak, moderate or strong positive activity. A total of seventeen *Vibrio* sp., *Bacillus* sp., *Ferrimonas* sp., and *Shewanella* sp. isolates exhibited weak positive protease activity, while six *Vibrio* sp. isolates and one *Bacillus* sp. strain showed moderate protease activity. On the other hand, five *Vibrio* sp. strains showed strong protease activity (Figure 4B).

### Biomineralization Ability

**Figure 5:**
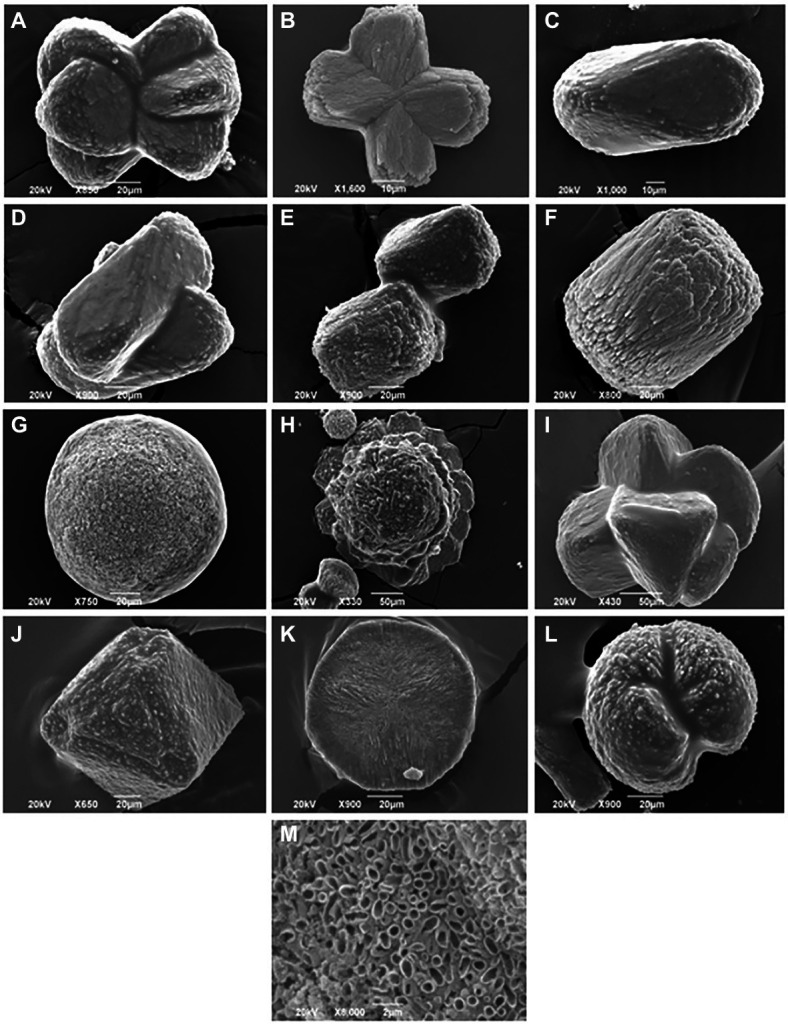
Scanning electron images of different crystals morphology harvested from sponge cultured bacteria. **(A)** Wheatsheaf shape, **(B)** Dumbbell shape, **(C)** Prism shape, **(D)** Intergrowth, **(E)** Cubic shape, **(F)** Cylinder shape, **(G)** Sphere shape, **(H)** Rosette shape, **(I)** Twinning shape, **(J)** Octahedrons shape, **(K)** Discoid shape, **(L)** Spherulite shape, and **(M)** close up image of disc shaped crystal.

In the present study, 11 out of 15 tested bacterial isolates cultivated from *Haliclona* sp. sponges showed the ability to precipitate calcium carbonate crystals. The calcium carbonate-producing isolates belonged to strains of *Bacillus* sp., *Shewanella* sp., *Pseudovibrio* sp. and *Vibrio* sp. As shown in Table 1 and Figure 5, two *Pseudovibrio* isolates generated crystals with a wide variety of shapes (*n* = 5–6 shapes from each isolate), such as sphere, dumbbell, prism, rosette, wheatsheaf, intergrowth, twinning and spherulite shapes. Four *Vibrio* sp. isolates produced both octahedrons and cylinder-shaped crystals. In addition to these shapes, each *Vibrio* strain generated additional crystal shapes, such as rosette-, cubic-, dumbbell- or prism-shaped crystals. Unlike the other strains, one *Vibrio* sp. isolate generated both dumbbell- and prism-shaped crystals. Furthermore, three *Bacillus* sp. isolates produced a total of 3 to 4 different crystal shapes, while *Shewanells* sp. presented the lowest crystal diversity (showing only sphere and spherulite shapes).

**Table 1:** Different shapes of calcium carbonate crystal produced by bacteria.

Crystal morphology	Isolate name/code										
	*Bacillus* sp. KS46	*Bacillus* sp. KS48	*Bacillus* sp. KS49	*Shewanella* sp. KS1	*Pseudovibrio* sp. KS8	*Pseudovibrio* sp. KS33a1	*Vibrio* sp. KS2	*Vibrio* sp. KS7	*Vibrio* sp. KS23	*Vibrio* sp. KS17	*Vibrio* sp. KS12h1
Sphere		X		X	X	X					
Dumbbell					X	X					X
Discoid	X										
Prism		X	X		X	X				X	X
Rosette	X				X				X		
Octahedrons		X					X	X	X	X	
Cylinder	X						X	X	X	X	
Cubic								X			
Wheatsheaf						X					
Intergrowth			X			X					
Twinning			X			X					
Spherulite				X	X						

## Discussion

In recent years, researchers have focused their interest on sponge microbial communities due to their importance in biotechnology (see full review by De Oliveira et al., 2020). There are few available studies on sponge-associated bacteria in the Arabian Gulf, and most of these studies were conducted in the north and northeastern regions of the Gulf. Najafi et al. (2018) used tag pyrosequencing to investigate bacteria associated with sponge samples and they were the first in the Gulf to evaluate the microbial community associated with *Suberites diversicolor*, *Pseudoceratina Arabica*, *Chondrilla* sp., *Cladocroce* sp., *Halichondria* sp., *Dictyoceratida* sp., and *Ircinia ramose* from an offshore site in Bushehr, Iran. Their research showed that the most abundant bacterial phyla associated with their sponge samples were Cyanobacteria, followed by Proteobacteria, Chloroflexi, Acidobacteria, and Actinobacteria. The remaining studies regarding sponge-associated microorganisms in the Arabian Gulf focused on their potential applications in biotechnology using either direct sponge extract or few isolates (7–12 isolates) associated with the tested sponge samples. A recent study from the west coast of the Arabian Gulf, specifically from a hyperarid mangrove region in Qatar, investigated the antimicrobial activity of two sponge samples (Giraldes et al., 2020). On the other hand, a wide range of culturable sponge-associated microorganisms (or sponge crude extract) from coast of Iran with potential biotechnological applications were investigated to assess their antimicrobial abilities, bio-degradation potential and cytotoxic properties (Safaeian et al., 2009; Khakshoor and Pazooki, 2014; Nazemi et al., 2014a,b; Pazooki and Khakshoor, 2015; Salimi et al., 2015; Ebadi and Sanati, 2016; Zarei et al., 2017; Karimpoor et al., 2018; Shushizadeh et al., 2018). Furthermore, only one study has been performed on sponges in Kuwait where Orabi (2011) examined the antimicrobial activity of sponge macerates. Therefore, the current study focuses on utilizing culture-dependent and culture-independent techniques to identify sponge-associated bacteria and investigate their potential biotechnological applications in the northwestern region of the Arabian Gulf.

In the current study, a rarefaction plot of 16S rRNA next-generation sequencing results showed that a sufficient number of reads were analyzed from seven sponge samples. The total number of OTUs varied among individual sponges of the same genus, such as *Niphates* sp. 1 and sp. 3, and among individuals of the same species, such as the examined *Haliclona* sp. samples (Supplementary Table S5). It should be noted that the data for sponge bacteriome using culture independent techniques are considered descriptive and not made for comparison due to the limitation in the number of processed samples. Previous studies used culture independent techniques from different locations to investigate and determine bacterial phyla associated with different sponge genera. These studies proved that phyla Proteobacteria is considered the most dominant phyla associated with their tested samples. For instance, Steinert et al. (2019) proved that Bacteroidetes, Proteobacteria (alpha, beta, gamma and delta) and Actinobacteria were abundant taxa associated with *Haliclona* sp., *Antarctotetilla leptoderma*, *Homaxinella balfourensis* and *Isodictya bentarti* sponges from the Antarctic. Cleary et al. (2019) reported a high relative abundance of Proteobacteria (Gammaproteobacteria) associated with the sponges *Haliclona cymaeformis* and *Xestospongia testudinari* from the Indo-Pacific. Furthermore, extensive research involving a dataset of 3,569 sponge specimens collected from several locations worldwide in “The Sponge Microbiome Project” assigned the majority of the processed OTUs to Proteobacteria (Moitinho-Silva et al., 2017); however, the project did not include any sponges from the Arabian Gulf. Despite the abovementioned limitation in the current study, phyla Proteobacteria (alpha, beta, and gamma), Firmicutes, Bacteroidetes, and Actinobacteria and novel sequences assigned to “unknown” group were the major phyla in the Kuwait sponge. Furthermore, additional phyla (Cyanobacteria, Planctomycetes, Dependentiae, Chlamydiae, Acidobacteria, Gemmatimonadetes, Verrucomicrobia, Chloroflexi, and Nitrospirae) dominated some, but not all, sponge samples. Future studies are needed to explore some unique sequences, such as the one labeled as unknown.

Our research aimed to combine culture-dependent and culture-independent techniques to investigate sponge-associated bacteria. The culture-independent technique was employed to describe the microbial communities associated with our sponge samples. The culture-dependent technique allowed the cultivation of only a small fraction of bacteria, as such techniques are highly selective and limited by the culture conditions and media type. It is estimated that 99% of sponge-associated bacteria are unculturable (Kennedy et al., 2008); nevertheless, culture-dependent techniques enable the exploration of potential biotechnological applications of cultivated bacterial isolates. The current study’s result supports that only a fraction of the bacteria associated with the sponge tissue is culturable. Using one media (i.e., Marine Agar) limited the diversity of the obtained culturable bacteria, but the percentage would remain limited to 1% even if more media were used. Nevertheless, interested strains were retrieved that were allowed to be explored.

In the current study, *Vibrio* (Gammaproteobacteria) and *Bacillus* (Firmicutes), followed by strains of *Shewanella*, *Ferrimonas*, *Pseudovibrio*, and *Spongiobacter* dominated *Haliclona* sp. 1Kl tissue. Previous studies have reported the cultivation of similar bacterial genera. For example, Kennedy et al. (2009) cultivated bacterial isolates belonging to the genera *Pseudovibrio* and *Bacillus* along with other bacterial strains from *Haliclona simulans*. Using a matrix of cultivation methods, Sipkema et al. (2011) cultivated isolates from *Haliclona* (*gellius*) sp. and found that 89% of the isolates were affiliated with Alphaproteobacteria, 5% with Gammaproteobacteria, 4% Bacteriodetes and less than 2% with other phyla. Other studies have reported the isolation of *Bacillus*, *Pseudovibrio*, *Spongiobacter*, *Shewanella,* and *Vibrio* from *Suberites carnous* and *Leucosolenia* sp. (Flemer et al., 2012), *Arenosclera brasiliensis* (Rua et al., 2014), *Pione* (*cliona*) *cf. vastifica* and *Siphonochalina siphonella* (Bibi et al., 2018) and *Xestospongia muta* and *Xestospongia testudinaria* (Montalvo et al., 2014). Despite that, all isolates are essential and worth studying; *Vibrio*, in particular, is frequently cultivated in Kuwait’s marine environment, and further investigation is required to understand its role in the sponge.

The results of the study showed that among a group of 53 isolates, seven different *Bacillus* strains exhibited various degrees of growth inhibition against indicator microorganisms. The production of secondary metabolites is important in marine animals, including sponges, as they are subjected to intensive evolutionary pressure from poisons, infection, predation and competition in their environments; therefore, the production of an arsenal of chemical defenses, such as secondary metabolites with antimicrobial activities (Thakur and Müller, 2004), is necessary in these sessile animals. Studies have proven the ability of sponge-associated bacteria from different sponge species to produce secondary metabolites with antimicrobial activity. For instance, a study proved that over 50% of the tested bacterial isolates cultivated from *H. simulans* sponges presented positive antimicrobial activity (Kennedy et al., 2009). In addition, Santos et al. (2010) obtained bacterial isolates from the sponges *Clathrina aurea, Haliclona* sp., *Mycale microsigmatosa, Paraleucilla magna, Petromica citrina, Dragmacidon reticulatus, Geodia corticostylifera, Polymastia janeirensis* and *Tedania ignis,* among which 9% of these isolates showed positive antimicrobial activity, and two of the most active isolated bacterial strains belonged to the genera *Bacillus* and *Pseudomonas*. Another study proved that bacterial isolates with positive antimicrobial activity were more abundant in Alphaproteobacteria and *Pseudoalteromonas* (Gammaproteobacteria; Hentschel et al., 2001). Although the isolated *Bacillus* strains, as shown in the result (Figure 4), did not exhibit potent antimicrobial activity against the indicator organisms, additional activities could be revealed by testing them against a wide range of microbes, such as other bacterial and fungal strains, viruses, and perhaps cancer cells.

In addition to antimicrobial activity, Figure 4B shows that 58% of the bacterial isolates have positive protease activity in which *Vibrio* strains presented different degrees of activity ranging from weak to strong; on the other hand, *Ferrimonas* sp., *Shewanella* sp., and *Bacillus* sp. strains showed weak protease activities. The production of hydrolytic enzymes, such as proteases, by sponge-associated bacteria is necessary to degrade organic substrates to facilitate the feeding process and to regenerate nutrients surrounding the sponge so that they can be utilized (Shanmughapriya et al., 2009). In biotechnology, protease enzymes play important roles in the manufacturing of foods, pharmaceuticals, and textiles (Wiseman, 1985). The production of protease enzymes by sponge-associated bacteria was investigated by Mohapatra et al. (2003), who studied eight sedentary marine organisms, including six sponges (*Aaptos* sp., *Spirastrella* sp. *Ircinia* sp., *Phyllospongia* sp., *Azorica* sp. and *Axinella* sp.). Their research proved that 61% of the sponge-associated bacteria produced protease enzymes. The enzyme-producing strains were distributed among bacterial groups affiliated with the phyla Proteobacteria, Firmicutes, Actinobacteria, and Bacteroidetes. Similar to our results, the enzyme-producing bacterial genera included strains of *Bacillus and Vibrio*.

The results of this study further showed the ability of several bacterial strains to carry on biomineralization and to precipitate calcium carbonate crystals with different shapes. Biomineralization is a process where living organisms produce minerals such as silicate in diatoms (Brunner et al., 2009), phosphates in vertebrates (Kawasaki et al., 2009) and carbonates in invertebrates (Clark, 2020). This process could occur *via* biologically induced mineralization (Lowenstam, 1981) and biologically controlled mineralization (Mann, 1983). Microbially induced calcium carbonate precipitation is a chemical process governed by the calcium concentration, dissolved inorganic carbon concentration, pH, and the availability of nucleation sites (Hammes and Verstraete, 2002). The precipitation of calcium carbonate generates crystals that vary in shape and size according to their chemical composition and level of maturation. The available results regarding the biomineralization ability of sponge-associated bacteria were directly observed *via* the microscopic examination of the crystals in sponge tissue. Vacelet et al. (1987) first reported the presence of irregular calcareous bodies in *Hemimycale* sponges. Furthermore, Uriz et al. (2012) suggested an exoskeletal function of calcifying bacteria, as she observed the accumulation of calcite spherules produced by endosymbiotic calcifying bacteria at the sponge periphery. In addition, a study by Garate et al. (2015) achieved the isolation of calcibacteria spherules from the sponge *Hemimycale columella*and found that the spherules formed a subectosomal cortex layer resembling a rudimentary exoskeleton. Garate et al. (2017) further expanded their research and used molecular analysis to investigate sponge-associated calcifying bacteria. When the spherules were extracted, they found that these bacteria belonged to the Alphaproteobacteria SAR116 clade. It is difficult to compare our results with data from previous studies since previous studies did not report the cultivation of sponge-associated bacteria with biomineralization abilities, nor did they report the different shapes of the calcium carbonate crystals produced by sponge-associated bacteria under laboratory conditions. Our study’s results further showed several bacterial strains’ ability to precipitate calcium carbonate crystals of different shapes in which the most dominant were cylinders, octahedrons, spheres, and prisms. Previous studies focused on observing calcium carbonate crystals in sponge tissues *in vivo*, while our data is the first to describe these crystals *in vitro*. Extensive studies are needed to understand calcium carbonate crystals precipitated by sponge-associated bacteria outside the host and under laboratory conditions.

Sponge-calcifying bacteria can be seen as low-cost builders of a sponge exoskeleton that helps the host repel potential predators, rather than just producers of secondary metabolites (Garate et al., 2015). Among our scanning electron microscopy images of calcium carbonate crystals, a magnified image of a disc-shaped crystal showed that some strains precipitate calcium carbonate around their cells (Figure 5M). A previous study used fluorescence *in situ* hybridization and transmission electron microscopy detected calcifying bacteria enveloped by 100 nm-thick calcites in sponge tissue (Uriz et al., 2012; Garate et al., 2017). Impressively, the calcification process within the calcibacteriocytes (i.e., calcifying bacteria contained within vacuoles in amoeboid archeocytes-like cells) can be maintained under control (Uriz et al., 2012) where calcibacteriocytes migrate to the sponge periphery to release calcified calcibacteria, leading to the calcareous layer formation. Such process is similar to archeocytes releasing bioactive metabolites near sponge surface in order to prevent sponge self-toxicity (Uriz et al., 1996, 2012; Garate et al., 2017). To our knowledge, no previous studies isolated calcifying bacteria *in vitro* from any marine organism. More research is needed to link the isolates with biomineralization ability with the formation of the calcareous crystals in the sponge periphery. We suspect that studies for other calcareous marine organisms such as corals may reveal more information about the role of the calcifying bacteria in marine animals’ holobionts.

## Conclusion

Our research has advanced knowledge of sponges on the northwestern coast of the Arabian Gulf and their associated microbiomes by using amplicon next-generation sequencing. This method detected more than 25 bacterial phyla associated with novel sponge species. Culture-dependent techniques allowed us to cultivate a total of 315 bacterial isolates associated with *Haliclona* sp. 1Kl and investigate their applications in biotechnology. Selected bacterial strains of the genera *Vibrio*, *Bacillus*, *Pseudovibrio,* and *Shewanella* induced the production of different shapes of calcium carbonate crystals under laboratory conditions. These results suggest the importance of sponge-associated bacteria in biotechnology. To the best of our knowledge, our research is the first to investigate sponge-associated bacteria in Kuwait and the first to demonstrate the isolation of sponge-associated calcifying bacteria and to harvest calcium carbonate crystals. Extensive research will ultimately be needed to explore marine sponges and improve the cultivation of their associated bacteria to further explore their potentially important biotechnological applications.

## Data Availability Statement

The data presented in the study are deposited in NCBI, accession number MK558635-MK558695 and PRJNA540061.

## Author Contributions

SA and HM: writing—original draft, review, and editing. SA: investigation. HM: formal analysis and resources. All authors contributed to the article and approved the submitted version.

## Funding

This research was sponsored by the Kuwait University, College of Graduate Studies.

## Conflict of Interest

The authors declare that the research was conducted in the absence of any commercial or financial relationships that could be construed as a potential conflict of interest.

## Publisher’s Note

All claims expressed in this article are solely those of the authors and do not necessarily represent those of their affiliated organizations, or those of the publisher, the editors and the reviewers. Any product that may be evaluated in this article, or claim that may be made by its manufacturer, is not guaranteed or endorsed by the publisher.

## Footnotes

^1^ https://github.com/torognes/vsearch

^2^ http://blast.ncbi.nlm.nih.gov/Blast.cgi

^3^ http://www.arb-silva.de

## References

[ref1] Becerro M. A. Lopez N. I. Turon X. Uriz M. J. (1994). Antimicrobial activity and surface bacterial film in marine sponges. J. Exp. Mar. Biol. Ecol. 179, 195–205. doi: 10.1016/0022-0981(94)90114-7

[ref2] Bibi F. Alvi S. A. Al-Sofyani A. Yasir M. Kensarah E. A. Azhar E. I. (2018). Two marine sponges-associated cultivable bacteria: diversity and biological activities. Genet. Mol. Res. 17:gmrl6039910. doi: 10.4238/gmr16039910

[ref3] Boquet E. Boronat A. Ramos-Cormenzana A. (1973). Production of calcite (calcium carbonate) crystals by soil bacteria is a general phenomenon. Nature 246, 527–529. doi: 10.1038/246527a0

[ref4] Boury-Ensnault N. Rützler K. (1997). Thesaurus of sponge morphology. Smithsonian Contrib. Zoo. 596, 1–55. doi: 10.5479/si.00810282.596

[ref5] Brunner E. Gröger C. Lutz K. Richthammer P. Spinde K. Sumper M. (2009). Analytical studies of silica biomineralization: towards an understanding of silica processing by diatoms. Appl. Microbiol. Biotechnol. 84, 607–616. doi: 10.1007/s00253-009-2140-3, PMID: 19629468

[ref6] Bull A. T. Stach J. E. (2007). Marine actinobacteria: new opportunities for natural product search and discovery. Trends Microbiol. 15, 491–499. doi: 10.1016/j.tim.2007.10.004, PMID: 17997312

[ref7] Camacho C. Coulouris G. Avagyan V. Ma N. Papadopoulos J. Bealer K.. (2009). BLAST plus: architecture and applications. BMC Bioinform. 10, 421–430. doi: 10.1186/1471-2105-10-421, PMID: 20003500 PMC2803857

[ref8] Chanas B. Pawlik J. R. Lindel T. Fenical W. (1997). Chemical defense of the Caribbean sponge *Agelas clathrodes* (Schmidt). J. Exp. Mar. Biol. Ecol. 208, 185–196. doi: 10.1016/S0022-0981(96)02653-6

[ref9] Clark M. S. (2020). Molecular mechanisms of biomineralization in marine invertebrates. J. Exp. Biol. 223:jeb206961. doi: 10.1242/jeb.206961, PMID: 32471885 PMC7272335

[ref10] Cleary D. F. Swierts T. Coelho F. J. Polónia A. R. Huang Y. M. Ferreira M. R.. (2019). The sponge microbiome within the greater coral reef microbial meta community. Nat. Commun. 10, 1–12. doi: 10.1038/s41467-019-09537-8 30967538 PMC6456735

[ref11] De Oliveira B. F. R. Carr C. M. Dobson A. D. Laport M. S. (2020). Harnessing the sponge microbiome for industrial biocatalysts. Appl. Microbiol. Biotechnol. 104, 8131–8154. doi: 10.1007/s00253-020-10817-3, PMID: 32827049

[ref12] Ebadi K. Sanati A. M. (2016). Biodegradation of crude oil with associated Bacteria isolated from the native sponge of the Persian Gulf Dictyonella sp. J. Oceanogr. 7, 59–68.

[ref13] Engel S. Pawlik J. R. (2000). Allelopathic activities of sponge extracts. Mar. Ecol. Prog. Ser. 207, 273–281. doi: 10.3354/meps207273

[ref14] Esteves A. I. Cullen A. Thomas T. (2017). Competitive interactions between sponge-associated bacteria. FEMS Microbiol. Ecol. 93:fix008. doi: 10.1093/femsec/fix008 28115399

[ref16] Flemer B. Kennedy J. Margassery L. M. Morrissey J. P. O’Gara F. Dobson A. D. W. (2012). Diversity and antimicrobial activities of microbes from two Irish marine sponges, Suberites carnosus and Leucosolenia sp. J. Appl. Microbiol. 112, 289–301. doi: 10.1111/j.1365-2672.2011.05211.x, PMID: 22129274

[ref17] Garate L. Blanquer A. Uriz M. J. (2015). Calcareous spherules produced by intracellular symbiotic bacteria protect the sponge Hemimycale columella from predation better than secondary metabolites. Mar. Ecol. Prog. Ser. 523, 81–92. doi: 10.3354/meps11196

[ref18] Garate L. Sureda J. Agell G. Uriz M. J. (2017). Endosymbiotic calcifying bacteria across sponge species and oceans. Sci. Rep. 7:43674. doi: 10.1038/srep43674, PMID: 28262822 PMC5337934

[ref19] Giles E. C. Kamke J. Moitinho-Silva L. Taylor M. W. Hentschel U. Ravasi T.. (2013). Bacterial community profiles in low microbial abundance sponges. FEMS Microbiol. Ecol. 83, 232–241. doi: 10.1111/j.1574-6941.2012.01467.x, PMID: 22882238

[ref20] Giraldes B. W. Goodwin C. Al-Fardi N. A. A. Engmann A. Leitão A. Ahmed A. A.. (2020). Two new sponge species (Demospongiae: Chalinidae and Suberitidae) isolated from hyperarid mangroves of Qatar with notes on their potential antibacterial bioactivity. PLoS One 15, 1–12. doi: 10.1371/journal.pone.0232205 PMC7219822 32401792

[ref21] Graça A. P. Viana F. Bondoso J. Correia M. I. Gomes L. Humanes M.. (2015). The antimicrobial activity of heterotrophic bacteria isolated from the marine sponge *Erylus deficiens* (Astrophorida, Geodiidae). Front. Microbiol. 6:389. doi: 10.3389/fmicb.2015.00389 25999928 PMC4423441

[ref22] Hammes F. Verstraete W. (2002). Key roles of pH and calcium metabolism in microbial carbonate precipitation. Rev. Environ. Sci. Biotechnol. 1, 3–7. doi: 10.1023/A:1015135629155

[ref23] Han B. N. Hong L. L. Gu B. B. Sun Y. T. Wang J. Liu J. T.. (2019). “Natural products from sponges,” in Symbiotic Microbiomes of Coral Reefs Sponges and Corals. ed. Li Z. (Berlin, Germany: Springer), 329–463.

[ref24] Hentschel U. Schmid M. Wagner M. Fieseler L. Gernert C. Hacker J. (2001). Isolation and phylogenetic analysis of bacteria with antimicrobial activities from the Mediterranean sponges *Aplysina aerophoba* and *Aplysina cavernicola*. FEMS Microbiol. Ecol. 35, 305–312. doi: 10.1111/j.1574-6941.2001.tb00816.x, PMID: 11311441

[ref25] Hoffmann F. Larsen O. Thiel V. Rapp H. T. Pape T. Michaelis W.. (2005). An anaerobic world in sponges. Geomicrobiol J. 22, 1–10. doi: 10.1080/01490450590922505

[ref26] Hooper J. N. Van Soest R. W. (2002). “A guide to the classification of Sponges,” in Systema Porifera. eds. Hooper J. N. A. Soest R. W. M. Willenz P. (United States: Springer), 1–7.

[ref27] Ionescu D. Siebert C. Polerecky L. Munwes Y. Y. Lott C. Häusler S.. (2012). Microbial and chemical characterization of underwater fresh water springs in the Dead Sea. PLoS One 7:e38319. doi: 10.1371/journal.pone.0038319, PMID: 22679498 PMC3367964

[ref28] Isaacson D. M. Kirschbaum J. (1986). “Assays of antimicrobial substances,” in Manual of Industrial Microbiology and Biotechnology. eds. Demainand A. L. Solomon N. A. (Washington, DC: ASM), 410–435.

[ref29] Jackson S. A. Kennedy J. Morrissey J. P. O’Gara F. Dobson A. D. (2012). Pyrosequencing reveals diverse and distinct sponge-specific microbial communities in sponges from a single geographical location in Irish waters. Microb. Ecol. 64, 105–116. doi: 10.1007/s00248-011-0002-x, PMID: 22281804

[ref30] Karimpoor M. Kamrani E. Yousefzadi M. Nazemi M. (2018). Antibacterial and antioxidant potential of *Haliclona caerulea* extracts from Tidal Island Larak, Persian Gulf. Modares J. Biotechnol. 9, 347–353.

[ref31] Kawasaki K. Buchanan A. V. Weiss K. M. (2009). Biomineralization in humans: making the hard choices in life. Annu. Rev. Genet. 43, 119–142. doi: 10.1146/annurev-genet-102108-134242, PMID: 19659443

[ref32] Kennedy J. Baker P. Piper C. Cotter P. D. Walsh M. Mooij M. J.. (2009). Isolation and analysis of bacteria with antimicrobial activities from the marine sponge *Haliclona simulans* collected from Irish waters. Mar. Biotechnol. 11, 384–396. doi: 10.1007/s10126-008-9154-1, PMID: 18953608

[ref33] Kennedy J. Codling C. E. Jones B. V. Dobson A. D. Marchesi J. R. (2008). Diversity of microbes associated with the marine sponge, *Haliclona simulans*, isolated from Irish waters and identification of polyketide synthase genes from the sponge metagenome. Environ. Microbiol. 10, 1888–1902. doi: 10.1111/j.1462-2920.2008.01614.x, PMID: 18430018

[ref34] Khakshoor M. S. Pazooki J. (2014). Bactericidal and fungicidal activities of different crude extracts of *Gelliodes carnosa* (sponge, Persian gulf). Iran. J. Fish. Sci. 13, 776–784. doi: 10.22092/ijfs.2018.114394

[ref35] Klindworth A. Pruesse E. Schweer T. Peplies J. Quast C. Horn M.. (2013). Evaluation of general 16S ribosomal RNA gene PCR primers for classical and next-generation sequencing-based diversity studies. Nucleic Acids Res. 41:e1. doi: 10.1093/nar/gks808, PMID: 22933715 PMC3592464

[ref37] Kuo J. Yang Y. T. Lu M. C. Wong T. Y. Sung P. J. Huang Y. S. (2019). Antimicrobial activity and diversity of bacteria associated with Taiwanese marine sponge *Theonella swinhoei*. Ann. Microbiol. 69, 253–265. doi: 10.1007/s13213-018-1414-3

[ref39] Lee O. O. Wang Y. Yang J. Lafi F. F. Al-Suwailem A. Qian P. Y. (2011). Pyrosequencing reveals highly diverse and species-specific microbial communities in sponges from the Red Sea. ISME J. 5, 650–664. doi: 10.1038/ismej.2010.165, PMID: 21085196 PMC3105750

[ref40] Li C. W. Chen J. Y. Hua T. E. (1998). Precambrian sponges with cellular structures. Science 279, 879–882. doi: 10.1126/science.279.5352.879, PMID: 9452391

[ref41] Lowenstam H. A. (1981). Minerals formed by organisms. Science 211, 1126–1131. doi: 10.1126/science.7008198 7008198

[ref42] Luter H. M. Widder S. Botté E. S. Wahab M. A. Whalan S. Moitinho-Silva L.. (2015). Biogeographic variation in the microbiome of the ecologically important sponge, *Carteriospongia foliascens*. PeerJ 3:e1435. doi: 10.7717/peerj.1435, PMID: 26713229 PMC4690404

[ref43] Mahmoud H. M. (2015). Variations in the abundance and structural diversity of microbes forming biofilms in a thermally stressed coral reef system. Mar. Pollut. Bull. 100, 710–718. doi: 10.1016/j.marpolbul.2015.10.030, PMID: 26494248

[ref44] Maldonado M. Cortadellas N. Trillas M. I. Rutzler K. (2005). Endosymbiotic yeast maternally transmitted in a marine sponge. Biol. Bull. 209, 94–106. doi: 10.2307/3593127, PMID: 16260769

[ref45] Mann S. (1983). “Mineralization in biological systems,” in Inorganic Elements in Biochemistry. ed. Cowan J. (Berlin, Heidelberg: Springer), 125–174.

[ref47] Mazotto A. M. Couri S. Damaso M. C. Vermelho A. B. (2013). Degradation of feather waste by *Aspergillus Niger* keratinases: comparison of submerged and solid-state fermentation. Int. Biodeterior. Biodegrad. 85, 189–195. doi: 10.1016/j.ibiod.2013.07.003

[ref48] Mohapatra B. R. Bapuji M. Sree A. (2003). Production of industrial enzymes (amylase, carboxymethylcellulase and protease) by bacteria isolated from marine sedentary organisms. Acta Biotechnol. 23, 75–84. doi: 10.1002/abio.200390011

[ref49] Moitinho-Silva L. Nielsen S. Amir A. Gonzalez A. Ackermann G. Cerrano C.. (2017). The sponge microbiome project. Gigascience 6:gix077. doi: 10.1093/gigascience/gix077 PMC5632291 29020741

[ref50] Montalvo N. F. Davis J. Vicente J. Pittiglio R. Ravel J. Hill R. T. (2014). Integration of culture-based and molecular analysis of a complex sponge-associated bacterial community. PLoS One 9:e90517. doi: 10.1371/journal.pone.0090517, PMID: 24618773 PMC3949686

[ref51] Moreno-Pino M. Cristi A. Gillooly J. F. Trefault N. (2020). Characterizing the microbiomes of Antarctic sponges: a functional metagenomic approach. Sci. Rep. 10, 1–12. doi: 10.1038/s41598-020-57464-2 31959785 PMC6971038

[ref52] Müller W. E. Grebenjuk V. A. Le Pennec G. Schröder H. C. Brümmer F. Hentschel U.. (2004). Sustainable production of bioactive compounds by sponges-cell culture and gene cluster approach: a review. Mar. Biotechnol. 6, 105–117. doi: 10.1007/s10126-002-0098-6 15085406

[ref53] Muyzer G. De Waal E. C. Uitterlinden A. G. (1993). Profiling of complex microbial populations by denaturing gradient gel electrophoresis analysis of polymerase chain reaction-amplified genes coding for 16S rRNA. App. Environ. Microbiol. 59, 695–700. doi: 10.1128/aem.59.3.695-700.1993, PMID: 7683183 PMC202176

[ref54] Najafi A. Moradinasab M. Nabipour I. (2018). First record of microbiomes of sponges collected from the Persian Gulf, using tag pyrosequencing. Front. Microbiol. 9:1500. doi: 10.3389/fmicb.2018.01500, PMID: 30034382 PMC6043863

[ref55] Nazemi M. Motallebi Moghanjoghi A. A. Jamili S. Mashinchian A. Ghavam Mostafavi P. (2014a). Comparison of antibacterial activities of *Ircinia mutans* extracts in two different seasons from Kish Island, Persian gulf, Iran. Iranian J. Fisheries Sci. 13, 823–833.

[ref56] Nazemi M. Salimi M. A. Salimi P. A. Motallebi A. Jahromi S. T. Ahmadzadeh O. (2014b). Antifungal and antibacterial activity of *Haliclona* sp. from the Persian Gulf, Iran. J. Mycol. Med. 24, 220–224. doi: 10.1016/j.mycmed.2014.03.005, PMID: 24934592

[ref57] Nguyen M. T. Thomas T. (2018). Diversity, host-specificity and stability of sponge-associated fungal communities of co-occurring sponges. PeerJ 6:e4965. doi: 10.7717/peerj.4965, PMID: 29888140 PMC5991299

[ref58] Ondov B. D. Bergman N. H. Phillippy A. M. (2011). Interactive metagenomic visualization in a web browser. BMC Bioinform. 12, 385–393. doi: 10.1186/1471-2105-12-385, PMID: 21961884 PMC3190407

[ref60] Orabi Y. K. (2011). Search for new leads from marine macrofauna: collection from Kuwaiti Arabian gulf coast. Int. J. Pharm. Sci. 3, 228–232.

[ref61] Pazooki J. Khakshoor M. S. (2015). Evaluation of the anti-microbial properties of *Gelliodes carnosa* sponge alkaloid compounds antimicrobial properties of marine sponge. Int. J. Aquatic Sci. 6, 84–95.

[ref62] Proksch P. Ebel R. Edrada R. Riebe F. Liu H. Diesel A.. (2008). Sponge-associated fungi and their bioactive compounds: the Suberites case. Bot. Mar. 51, 209–218. doi: 10.1515/BOT.2008.014

[ref63] Proksch P. Edrada R. Ebel R. (2002). Drugs from the seas–current status and microbiological implications. Appl. Microbiol. Biotechnol. 59, 125–134. doi: 10.1007/s00253-002-1006-8 12111137

[ref64] Pruesse E. Peplies J. Glöckner F. O. (2012). SINA: accurate high-throughput multiple sequence alignment of ribosomal RNA genes. Bioinformatics 28, 1823–1829. doi: 10.1093/bioinformatics/bts252, PMID: 22556368 PMC3389763

[ref65] Quast C. Pruesse E. Yilmaz P. Gerken J. Schweer T. Yarza P.. (2013). The SILVA ribosomal RNA gene database project: improved data processing and web-based tools. Nucleic Acids Res. 41, D590–D596. doi: 10.1093/nar/gks1219 23193283 PMC3531112

[ref67] Rognes T. Flouri T. Nichols B. Quince C. Mahé F. (2016). VSEARCH: a versatile open source tool for metagenomics. PeerJ 4:e2584. doi: 10.7717/peerj.2584, PMID: 27781170 PMC5075697

[ref68] Rua C. P. Trindade-Silva A. E. Appolinario L. R. Venas T. M. Garcia G. D. Carvalho L. S.. (2014). Diversity and antimicrobial potential of culturable heterotrophic bacteria associated with the endemic marine sponge *Arenosclera brasiliensis*. PeerJ 2:e419. doi: 10.7717/peerj.419, PMID: 25024903 PMC4081303

[ref69] Safaeian S. Hosseini H. Asadolah A. A. P. Farmohamadi S. (2009). Antimicrobial activity of marine sponge extracts of offshore zone from nay Band Bay. J. Mycol. Méd. 19, 11–16. doi: 10.1016/j.mycmed.2008.11.003

[ref70] Salimi A. Saharkhiz M. P. Motallebi A. Seydi E. Mohseni A. R. Nazemi M.. (2015). Standardized extract of the Persian Gulf sponge, *Axinella sinoxea* selectively induces apoptosis through mitochondria in human chronic lymphocytic leukemia cells. J. Analitical Oncol. 4, 132–140. doi: 10.6000/1927-7229.2015.04.04.2

[ref71] Santos O. C. Pontes P. V. Santos J. F. Muricy G. Giambiagi-deMarval M. Laport M. S. (2010). Isolation, characterization and phylogeny of sponge-associated bacteria with antimicrobial activities from Brazil. Res. Microbiol. 161, 604–612. doi: 10.1016/j.resmic.2010.05.013, PMID: 20600863

[ref72] Schmitt S. Weisz J. B. Lindquist N. Hentschel U. (2007). Vertical transmission of a phylogenetically complex microbial consortium in the viviparous sponge *Ircinia felix*. Appl. Environ. Microbiol. 73, 2067–2078. doi: 10.1128/AEM.01944-06, PMID: 17277226 PMC1855684

[ref74] Shanmughapriya S. Kiran G. S. Selvin J. Gandhimathi R. Baskar T. B. Manilal A. (2009). Optimization, production, and partial characterization of an alkalophilic amylase produced by sponge-associated marine bacterium *Halobacterium salinarum* MMD047. Biotechnol. Bioprocess Eng. 14, 67–75. doi: 10.1007/s12257-008-0060-1

[ref75] Sharp K. H. Eam B. Faulkner D. J. Haygood M. G. (2007). Vertical transmission of diverse microbes in the tropical sponge Corticium sp. Appl. Environ. Microbiol. 73, 622–629. doi: 10.1128/AEM.01493-06, PMID: 17122394 PMC1796987

[ref76] Sheppard C. (2009). Large temperature plunges recorded by data loggers at different depths on an Indian Ocean atoll: comparison with satellite data and relevance to coral refuges. Coral Reefs 28, 399–403. doi: 10.1007/s00338-009-0476-x

[ref77] Shushizadeh M. R. Behroozi S. Behfar A. A. Nazemi M. (2018). Antibacterial activity and Gc-mass analysis of organic extract from Persian gulf *Haliclona* sp. Pharmacophore 9, 19–24.

[ref78] Simister R. L. Deines P. Botté E. S. Webster N. S. Taylor M. W. (2012). Sponge specific clusters revisited: a comprehensive phylogeny of sponge associated microorganisms. Environ. Microbiol. 14, 517–524. doi: 10.1111/j.1462-2920.2011.02664.x, PMID: 22151434

[ref79] Sipkema D. Schippers K. Maalcke W. J. Yang Y. Salim S. Blanch H. W. (2011). Multiple approaches to enhance the cultivability of bacteria associated with the marine sponge *Haliclona* (gellius) sp. Appl. Environ. Microbiol. 77, 2130–2140. doi: 10.1128/AEM.01203-10, PMID: 21296954 PMC3067343

[ref80] Staley J. T. Konopka A. (1985). Measurement of in situ activities of nonphotosynthetic microorganisms in aquatic and terrestrial habitats. Annu. Rev. Microbiol. 39, 321–346. doi: 10.1146/annurev.mi.39.100185.001541, PMID: 3904603

[ref81] Steinert G. Wemheuer B. Janussen D. Erpenbeck D. Daniel R. Simon M.. (2019). Prokaryotic diversity and community patterns in Antarctic continental shelf sponges. Front. Mar. Sci. 6:297. doi: 10.3389/fmars.2019.00297

[ref83] Sun W. Dai S. Jiang S. Wang G. Liu G. Wu H.. (2010). Culture-dependent and culture-independent diversity of Actinobacteria associated with the marine sponge *Hymeniacidon perleve* from the South China Sea. Antonie Van Leeuwenhoek 98, 65–75. doi: 10.1007/s10482-010-9430-8, PMID: 20383659

[ref84] Taylor M. W. Radax R. Steger D. Wagner M. (2007). Sponge-associated microorganisms: evolution, ecology, and biotechnological potential. Microbiol. Mol. Biol. Rev. 71, 295–347. doi: 10.1128/MMBR.00040-06, PMID: 17554047 PMC1899876

[ref85] Thakur N. L. Müller W. E. (2004). Biotechnological potential of marine sponges. Curr. Sci. 86, 1506–1512.

[ref86] Turon M. Uriz M. (2020). New insights into the Archaeal consortium of tropical sponges. Front. Mar. Sci. 6:789. doi: 10.3389/fmars.2019.00789

[ref87] Uriz M. J. Agell G. Blanquer A. Turon X. Casamayor E. O. (2012). Endosymbiotic calcifying bacteria: a new cue to the origin of calcification in metazoa? Evolution. Int J. Evolution 66, 2993–2999. doi: 10.1111/j.1558-5646.2012.01676.x, PMID: 23025593 PMC3485668

[ref88] Uriz M. J. Becerro M. A. Tur J. M. Turon X. (1996). Location of toxicity within the Mediterranean sponge *Crambe crambe* (Demospongiae: Poecilosclerida). Mar. Biol. 124, 583–590. doi: 10.1007/BF00351039

[ref89] Vacelet J. Donadey C. Froget C. (1987). “The calcium carbonate spherules of *Hemimycale columella* (Demosponges, Poecilosclerida) and their taxonomic value,” in Taxonomy of Porifera. eds. Vacelet J. Boury-Esnault N. (Berlin, Heidelberg: Springer), 259–274.

[ref90] Van Soest R. W. M. Boury-Esnault N. Hooper J. N. A. Rützler K. de Voogd N. J. Alvarez B.., (2018). World Porifera database. Available at: http://www.marinespecies.org/porifera (Accessed March 10, 2018).

[ref91] Van Soest R. W. Boury-Esnault N. Vacelet J. Dohrmann M. Erpenbeck D. De Voogd N. J.. (2012). Global diversity of sponges (Porifera). PLoS One 7:e35105. doi: 10.1371/journal.pone.0035105, PMID: 22558119 PMC3338747

[ref92] Varijakzhan D. Loh J. Y. Yap W. S. Yusoff K. Seboussi R. Lim S. E.. (2021). Bioactive compounds from marine sponges: fundamentals and applications. Mar. Drugs 19:246. doi: 10.3390/md19050246, PMID: 33925365 PMC8146879

[ref93] Villegas-Plazas M. Wos-Oxley M. L. Sanchez J. A. Pieper D. H. Thomas O. P. Junca H. (2019). Variations in microbial diversity and metabolite profiles of the tropical marine sponge *Xestospongia muta* with season and depth. Microb. Ecol. 78, 243–256. doi: 10.1007/s00248-018-1285-y, PMID: 30413836

[ref94] Weisz J. B. Hentschel U. Lindquist N. Martens C. S. (2007). Linking abundance and diversity of sponge-associated microbial communities to metabolic differences in host sponges. Mar. Biol. 152, 475–483. doi: 10.1007/s00227-007-0708-y

[ref95] Wilkinson C. R. (1983). Net primary productivity in coral reef sponges. Science 219, 410–412. doi: 10.1126/science.219.4583.410, PMID: 17815320

[ref96] Wilkinson C. R. Fay P. (1979). Nitrogen fixation in coral reef sponges with symbiotic cyanobacteria. Nature 279, 527–529. doi: 10.1038/279527a0

[ref97] Wiseman A. (1985). Handbook of Enzyme Biotechnology. New York: Ellis Horwood Ltd.

[ref98] Wulff J. (2001). Assessing and monitoring coral reef sponges: why and how? Bull. Mar. Sci. 69, 831–846.

[ref99] Yang J. Sun J. Lee O. O. Wong Y. H. Qian P. Y. (2011). Phylogenetic diversity and community structure of sponge-associated bacteria from mangroves of the Caribbean Sea. Aquat. Microb. Ecol. 62, 231–240. doi: 10.3354/ame01473

[ref100] Zarei M. Jahedi M. Nozhat F. (2017). Antibacterial activity of actinomycetes isolated from native sponge of Persian gulf (Kharku Island) against pathogens. J. Aquatic Ecol. 7, 50–58.

