# Supplementary material for: Identification of Sponge-Associated Bacteria From the Coast of Kuwait and Their Potential Biotechnological Applications
Source: Front Microbiol. 2022 Jul 4;13:896718. doi: 10.3389/fmicb.2022.896718 (PMC9289682; doi:10.3389/fmicb.2022.896718)
Supplement: Supplementary file 1 [file Data_Sheet_1.docx]

**Supplementary Materials**

**Table S1.** List of studies that isolated and identified sponge-associated bacteria using culture-dependent techniques.

**Table S2.** Culture characteristics of *Haliclona* sp. 1KI cultured bacteria.

**Table S3.** Culturable bacteria associated with *Haliclona* sp. 1KI and their nearest match from the GenBank (March 2020).

**Table S4.** Sampling site, date and taxonomic identification of sponge sample collected from Kuwait marine environment.

**Table S5.** The documented numbers of sponges-associated prokaryotes raw 16S rRNA amplicon next generation sequencing reads, prokaryotic operational taxonomic units (OTUs) and genera.

**Table S6.** Number of spots containing the filtered bacterial sequence (cultured bacterial isolates) found in *Haliclona* sp. NGS reads (SRX14840950).

**Table S7.** Antimicrobial activity of bacterial isolates obtained from in *Haliclona* sp. 1Kl.

**Figure S1.** Sponge samples collected from Kuwait marine environment and identified as (A) *Haliclona* sp., sample code 1KI, (B) *Haliclona* sp. sample code 5KI (C) *Chondrilla australiensis* sample code 4KI, (D) *Niphates sp.*1 sample code 2NW, (E) *Niphates sp.*2 sample code 5NW, (F) *Niphates sp.3* sample code 2KI, (G) *Amphimedon sp.* sample code 6KI. Sampling site and date: KI= Kubbar Island (August, 2017), NW= Nuwaiseeb intertidal (February, 2018).

**Figure S2.** Rarefaction curves of 16S rRNA gene diversity for 7 sponge sample collected from Kuwait.

**Table S1.** List of studies that isolated and identified sponge-associated bacteria using culture-dependent techniques.

| **Location** | **Sponge species** | **Types of culturing media** | **Selected and dominant bacterial phyla** | **Reference** |
| --- | --- | --- | --- | --- |
| Mediterranean Sea  Limski kanal  Banjole  St. Giovanni | *Chondrilla nucula, Tethya aurantium,*  *Suberites domuncula, Acanthella acuta*.  *Chondrosia reniformis*  *Clathrina clathris, Agelas oroides,*  *Ircinia* sp. | Semisynthetic polycarbon agar (HSPC), GPYNS agar, Chitin agar, Actinomycetes isolation agar (AIA), FS agar with 4 different YE concentration | Proteobacteria  (Alphaproteobacteria, Gammaproteobacteria), and Actinobacteria, | Muscholl-silberhorn et al. (2008) |
| South east India.  Vizhinjam coast | *Dendrilla nigra* | 14 different culturing media | Actinobacteria and Proteobacteria | Selvin et al. (2009) |
| Brazil  Cagarras Archipelago and Praia Vermelha beach | *Clathrina aurea, Dragmacidon reticulatus, Geodia corticostylifera, Haliclona sp., Mycale microsigmatosa, Paraleucilla magna, petromica citrina, Polymastia janeirensis and Tedania ignis* | Brain heart infusion (BHI) media, Marine Agar (MA), Marine agar in seawater (MSW) | Proteobacteria  (Alphaproteobacteria, Gammaproteobacteria), and Firmicutes | Santos et al. (2010) |
| United states of America  Monterey, California | *Haliclona* (*gellius*) sp. | agar plates (nineteen different culturing media),  Liquid media and floating filters. | Proteobacteria  (Alphaproteobacteria, Betaproteobacteria, Gammaproteobacteria and Deltaproteobacteria),  Bacteroidetes, Actinobacteria, Firmicutes, Verrucomicrobia, and Planctomycetes | Sipkema et al. (2011) |
| Southern coast of Portugal | *Sarcotragus spinosulus****,***  *Ircinia variabilis* | MA | Proteobacteria,  Bacteriodetes,  Firmicutes, and Actinobacteria | Esteves et al. (2013) |
| Eastern Mediterranean coast of Turkey  Beşparmak Island, Dana Island and Akkuyu Bay | *Ircinia variabilis, Petrosia ficiformis, Axinella damicornis, Dysidea avara, Agelas oroides, Sarcotragus spinosulus, Ircinia* sp., *Chondrosia reniformis, Sarcotragus fasciculatus, Phorbas fictitious, Axinella polypoides, Spirastrella cunctatrix,Axinella damicornis, Chondrilla nucula*, and *Oscarella lobularis.* | M1, M6, R2A, Actinomycetes isolation agar, SE medium (sponge extract), AW medium (agar water), ISP4 (with 6% NaCl), and SMP | A total of 7578 Actinomycetes colonies. | Öner et al. (2014) |
| Abra Harbor, Guam | *Rhabdastrella globostellata* | Diffusion growth chambers | Proteobacteria (Alpha and Gammaproteobacteria), Bacteroidetes, Actinobacteria and Firmicutes | Steinert et al., (2014) |
| Brazil  Comprida Island and Pargos Island | *Oscarella* spp | BHI and MA | Proteobacteria (Gammaproteobacteria) and Firmicutes (Bacilli) | Laport et al. (2017) |
| South Africa  Algoa Bay, Port Elizabeth. | *Isodictya compressa* and *Higginsia bidentifera* | 23 different culturing media | Actinobacteria, Firmicutes and Proteobacteria (Alphaproteobacteria and  Gammaproteobacteria). | Matobole et al. (2017) |
| Kenting National Park, southern Taiwan | *Theonella swinhoei* | Soil agar, Gause modified agar, actinomycete isolation agar, M1A agar, glucose-peptone-yeast extract agar, Gause mineral agar, and peptone-yeast extract agar. | Firmicutes, Actinobacteria, and Proteobacteria | Kuo et al. (2019) |
| South-west Iceland | *Halichondria panicea* | MA | Proteobacteria (Alpha and Gammaproteobacteria), Flavobacteriia, Planctomycetia, Verrucomicrobiae. | Knobloch et al. (2019) |
| Southeast coast of India | *Pirastrella inconstans*, *Cliona* sp., *Haliclona implexa*, *Callyspongia diffusa*, *Stylissa* sp., *Orina sagittaria*, *Tethya diploderma* and 2 unidentified sponges. | MA and seawater nutrient agar (SWNA). | Proteobacteria (Gammaproteobacteria), Firmicutes and Actinobacteria | Rajasabapathy et al. (2020) |

**
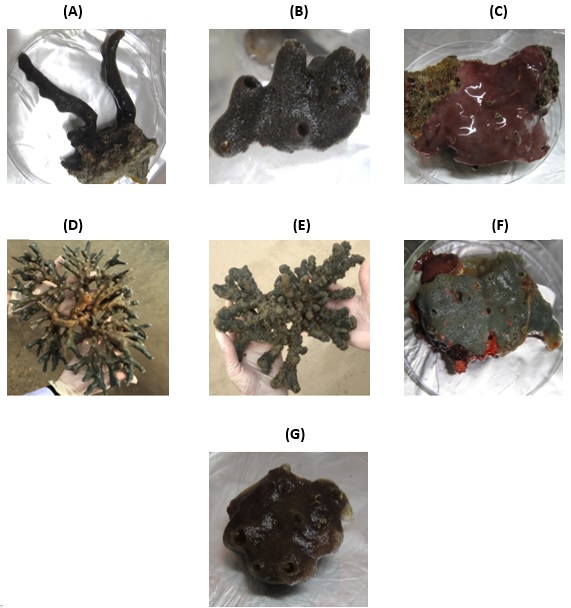
**

**Figure S1.** Sponge samples collected from Kuwait marine environment and identified as (A) *Haliclona* sp., sample code 1KI, (B) *Haliclona* sp. sample code 5KI (C) *Chondrilla australiensis* sample code 4KI, (D) *Niphates sp.*1 sample code 2NW, (E) *Niphates sp.*2 sample code 5NW, (F) *Niphates sp.3* sample code 2KI, (G) *Amphimedon sp.* sample code 6KI. Sampling site and date: KI= Kubbar Island (August, 2017), NW= Nuwaiseeb intertidal (February, 2018).

**
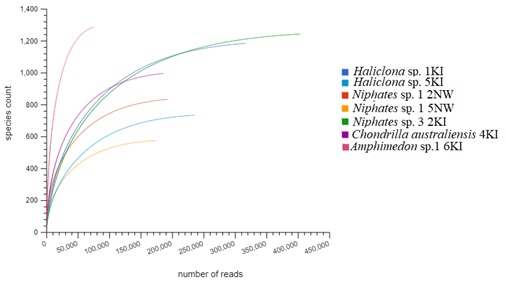
**

**Figure S2.** Rarefaction curves of 16S rRNA gene diversity for 7 sponge sample collected from Kuwait.

**Table S2.** Culture characteristics of *Haliclona* sp. 1KI cultured bacteria.

| **Isolate** | **Colony size** | **Color** | **Form** | **Margin** | **Elevation** | **Gram stain** | **Cell morphology** | **Cell size (µm)** | | | |
| --- | --- | --- | --- | --- | --- | --- | --- | --- | --- | --- | --- |
|  |  |  |  |  |  |  |  | **Length** | | | **Width** |
| KS55 | m | Beige | Circular | Entire | Convex | G-ve | Rod | 2 | | | 1 |
| KS59 | m | Beige | Irregular | Undulate | Flat | G-ve | Rod | 2 | | | 1 |
| KS58 | pp | Beige | Circular | Entire | Convex | G-ve | Rod | 4 | | | 0.8 |
| KS42 | m | Beige | Circular | Entire | Convex | G-ve | Rod | 2 | | | 1 |
| KS50 | s | Cream | Circular | Entire | Convex | G+ve | Rod | 3 | | 1 | |
| KS48 | s | Cream | Circular | Entire | Convex | G+ve | Rod | 3.5 | | 1 | |
| KS9 | m | Cream | Circular | Entire | Convex | G-ve | Coccobacilli | 1.5 | | 1 | |
| KS7 | m | Cream | Circular | Entire | Convex | G-ve | Coccobacilli | 1.4 | | 1 | |
| KS18 | pp | Cream | Circular | Entire | Raised | G-ve | Rod | 2.5 | | 1 | |
| KS30 | m | Cream | Circular | Entire | Convex | G-ve | Rod | 3 | 1 | | |
| KS26 | m | Cream | Circular | Entire | Convex | G-ve | Rod | 2.5 | 1 | | |
| KS27 | s | Cream | Circular | Entire | Raised | G-ve | Rod | 3 | 1 | | |
| KS28 | m | Cream | Circular | Entire | Convex | G-ve | Coccobacilli | 1.5 | 1 | | |
| KS38 | m | cream | circular | entire | convex | G-ve | Rod | 2 | 1 | | |
| KS44 | s | Cream | Circular | Entire | Convex | G-ve | Rod | 3 | 1 | | |
| KS54 | m | Translucent cream | Circular | Entire | Convex | G-ve | Rod | 2 | 1 | | |
| KS45 | s | Translucent Cream | Circular | Entire | Convex | G-ve | Rod | 2 | 1 | | |
| KS16 | s | Translucent Cream | Circular | Entire | Convex | G-ve | Rod | 2 | 1 | | |
| KS8 | pp | Translucent Cream | Circular | Entire | Convex | G-ve | Rod | 4 | 1 | | |
| KS36 | s | Translucent Cream | Circular | Entire | Convex | G-ve | Rod | 2 | 1 | | |
| KS37 | m | Translucent Cream | Circular | Entire | Convex | G-ve | Rod | 2 | 1 | | |
| KS34 | m | Translucent Cream | Circular | Entire | Convex | G-ve | Rod | 2 | 1 | | |
| KS52 | pp | Translucent Cream | Circular | Entire | Convex | G-ve | Rod | 3 | 1 | | |
| KS49 | s | Translucent Cream | Irregular | Undulate | Raised | G+ve | Rod | 2.8 | 0.8 | | |
| KS40 | m | Translucent cream | Circular | Entire | Convex | G-ve | Rod | 2.5 | 1 | | |
| KS61 | pp | Translucent Cream | Circular | Entire | Convex | G-ve | Rod | 2.5 | 1 | | |
| KS31 | s | Translucent Cream - pink | Circular | Entire | Convex | G-ve | Rod | 2 | 1 | | |
| KS13 | s | Translucent Cream -yellow | Circular | Entire | Convex | G-ve | Long Rod | 5 | 2 | | |
| KS3 | s | Translucent Cream -yellow | Circular | Entire | Convex | G-ve | Rod | 3 | 1.5 | | |
| KS4 | m | Translucent Cream -yellow | Circular | Entire | Convex | G-ve | Coccobacilli | 1.5 | 1 | | |
| KS10 | s | Translucent Cream -yellow | Circular | Entire | Convex | G-ve | Rod | 2.5 | 1 | | |
| KS22 | L | Translucent Cream -yellow | Circular | Entire | Convex | G-ve | Coccobacilli | 1.5 | 1 | | |
| KS19 | l | Translucent Cream -yellow | Circular | Entire | Convex | G-ve | Rod | 2 | 1.5 | | |
| KS2 | m | Translucent Cream -yellow | Circular | Entire | Convex | G-ve | Coccobacilli | 1.5 | 1 | | |
| KS17 | m | Translucent Cream -yellow | Circular | Entire | Convex | G-ve | Rod | 2 | 1 | | |
| KS32 | pp | Translucent Cream -yellow | Circular | Entire | Convex | G-ve | Rod | 2 | 1 | | |
| KS29 | s | Translucent Cream -yellow | Circular | Entire | Convex | G-ve | Rod | 2 | 1 | | |
| KS1 | s | Translucent Cream -orange | Circular | Entire | Convex | G-ve | Rod | 3 | 1.5 | | |
| KS6 | s | Translucent Cream -orange | Circular | Entire | Convex | G-ve | Long Rods | 5 | 0.8 | | |
| KS20 | s | Translucent Cream -orange | Circular | Entire | Convex | G-ve | Long threads | 12 | 0.5 | | |
| KS24 | s | Translucent Cream -orange | Irregular | Undulate | Raised | G-ve | Rod | 5 | 1 | | |
| KS12 | s | Translucent Cream -orange | Circular | Entire | Convex | G-ve | Rod | 4 | 1.5 | | |
| KS25 | m | Translucent Cream -orange | Circular | Entire | Convex | G-ve | Rod | 2 | 1 | | |
| KS11 | m | Translucent Cream -orange | Circular | Entire | Convex | G-ve | Rod | 2 | 1 | | |
| KS33 | s | Translucent Cream -orange | Circular | Entire | Convex | G-ve | Rod | 2 | 1 | | |
| KS5 | s | Translucent | Irregular | Undulate | Raised | G-ve | Long Rods | 6 | 0.5 | | |
| KS15 | s | Translucent | Irregular | Undulate | Raised | G-ve | Long threads | 12 | 0.4 | | |
| KS41 | s | Translucent | Circular | Entire | Convex | G-ve | Long Rod | 6 | 1.5 | | |
| KS51 | m | Yellow | Circular | Entire | Convex | G+ve | Rod | 3 | 1.5 | | |
| KS53 | s | White | Circular | Entire | Convex | G-ve | Rod | 2 | 1 | | |
| KS47 | s | White | Irregular | Undulate | Raised | G+ve | Rod | 4 | 2 | | |
| KS57 | m | White | Circular | Entire | Convex | G-ve | Rod | 3 | 1 | | |
| KS14 | m | White | Circular | Entire | Convex | G-ve | Rod | 2.5 | 1.2 | | |
| KS39 | m | White | Circular | Entire | Convex | G-ve | Rod | 2.5 | 1 | | |
| KS46 | s | White | Circular | Entire | Convex | G+ve | Rod | 3 | 1 | | |
| KS60 | s | White | Circular | Entire | Raised | G+ve | Rod | 4 | 0.8 | | |
| KS56 | s | Orange | Circular | Entire | Raised | G+ve | Rod | 2 | 1 | | |
| KS35 | m | Orange | Circular | Entire | Convex | G-ve | Rod | 2 | 1 | | |

KS: Kuwait sponge. s: small. vpp: very pinpoint.

m: medium. pp: pinpoint. l: large.

**Table S3.** Culturable bacteria associated with *Haliclona* sp. 1KI and their nearest match from the GenBank (data retrieved from NCBI on March 2020).

| **Accession No.** | **E-value** | **Similarity %** | **Nearest GenBank match** | **Phylum** | **Base compared** | **Total base pair** | **Isolate name** |
| --- | --- | --- | --- | --- | --- | --- | --- |
| MG972446.1 | 0.0 | 100 | *Shewanella corallii* strain JKB2 | Proteobacteria | 487/487 | 487 | *Shewanella* sp. KS1 |
| MT269637.1 | 0.0 | 99 | *Vibrio brasiliensis* strain DS1907-1YS_1_2 | Proteobacteria | 486/490 | 490 | *Vibrio* sp. KS2 |
| CP031472.1 | 0.0 | 100 | *Vibrio coralliilyticus* strain RE22 | Proteobacteria | 465/465 | 465 | *Vibrio* sp. KS3 |
| CP031472.1 | 0.0 | 99 | *Vibrio coralliilyticus* strain RE22 | Proteobacteria | 482/484 | 484 | *Vibrio* sp. KS4 |
| AB682172.1 | 0.0 | 99 | *Ferrimonas* sp. NBRC 104252 | Proteobacteria | 490/496 | 496 | *Ferrimonas* sp. KS5 |
| AB682172.1 | 0.0 | 99 | *Ferrimonas* sp. NBRC 104252 | Proteobacteria | 483/487 | 487 | *Ferrimonas* sp. KS6 |
| JN871709.1 | 0.0 | 99 | *Vibrio brasiliensis* strain FD W9 | Proteobacteria | 490/497 | 497 | *Vibrio* sp. KS7 |
| MH283837.1 | 0.0 | 100 | *Pseudovibrio denitrificans* strain SCSIO_43753 | Proteobacteria | 442/442 | 442 | *Pseudovibrio* sp. KS8 |
| KX418475.1 | 0.0 | 99 | *Vibrio harveyi* strain 50161 | Proteobacteria | 493/495 | 495 | *Vibrio* sp. KS9 |
| JN871710.1 | 0.0 | 99 | *Vibrio sinaloensis* strain FD O5-12 | Proteobacteria | 498/502 | 502 | *Vibrio* sp. KS10 |
| MK308628.1 | 0.0 | 99 | *Vibrio coralliilyticus* strain SC53 | Proteobacteria | 485/488 | 488 | *Vibrio* sp. KS11 |
| KY655377.1 | 0.0 | 99 | *Spongiobacter* sp. strain EA276 | Proteobacteria | 490/493 | 493 | *Spongiobacter* sp. KS12 |
| KY655377.1 | 0.0 | 99 | *Spongiobacter* sp. strain EA276 | Proteobacteria | 497/498 | 498 | *Spongiobacter* sp. KS13 |
| MF125195.1 | 0.0 | 99 | *Vibrio azureus* strain WUDI8 | Proteobacteria | 497/499 | 499 | *Vibrio* sp. KS14 |
| AB682172.1 | 0.0 | 99 | *Ferrimonas* sp. NBRC 104252 | Proteobacteria | 470/475 | 475 | *Ferrimonas* sp. KS15 |
| MF417434.1 | 0.0 | 100 | *Shewanella corallii* strain fav-2-10-05 | Proteobacteria | 493/493 | 493 | *Shewanella* sp*.* KS16 |
| MF417426.1 | 0.0 | 100 | *Vibrio sinaloensis* strain F17C1 | Proteobacteria | 465/470 | 470 | *Vibrio* sp. KS17 |
| MG972446.1 | 0.0 | 100 | *Shewanella corallii* strain JKB2 | Proteobacteria | 495/495 | 495 | *Shewanella* sp*.* KS18 |
| MF580374.1 | 0.0 | 99 | *Vibrio jasicida* strain OF-GM70 | Proteobacteria | 473/480 | 480 | *Vibrio* sp. KS19 |
| AB682172.1 | 0.0 | 99 | *Ferrimonas* sp. NBRC 104252 | Proteobacteria | 490/494 | 494 | *Ferrimonas* sp. KS20 |
| MH283837.1 | 0.0 | 100 | *Pseudovibrio denitrificans* strain SCSIO_43753 | Proteobacteria | 475/475 | 475 | *Pseudovibrio* sp. KS21 |
| JN871710.1 | 0.0 | 99 | *Vibrio sinaloensis* strain FD O5-12 | Proteobacteria | 487/491 | 491 | *Vibrio* sp. KS22 |
| CP031472.1 | 0.0 | 100 | *Vibrio coralliilyticus* strain RE22 | Proteobacteria | 485/485 | 485 | *Vibrio* sp. KS23 |
| AB682172.1 | 0.0 | 99 | *Ferrimonas* sp. NBRC 104252 | Proteobacteria | 488/493 | 493 | *Ferrimonas* sp. KS24 |
| CP031472.1 | 0.0 | 100 | *Vibrio coralliilyticus* strain RE22 | Proteobacteria | 482/482 | 482 | *Vibrio* sp. KS25 |
| CP033138.1 | 0.0 | 99 | *Vibrio owensii* strain 1700302 | Proteobacteria | 494/496 | 496 | *Vibrio* sp. KS26 |
| CP033138.1 | 0.0 | 100 | *Vibrio owensii* strain 1700302 | Proteobacteria | 479/479 | 479 | *Vibrio* sp. KS27 |
| CP033138.1 | 0.0 | 100 | *Vibrio owensii* strain 1700302 | Proteobacteria | 479/479 | 479 | *Vibrio* sp. KS28 |
| CP031472.1 | 0.0 | 100 | *Vibrio coralliilyticus* strain RE22 | Proteobacteria | 482/482 | 482 | *Vibrio* sp. KS29 |
| MK318661.1 | 0.0 | 100 | *Vibrio harveyi* strain 2018MZ1 | Proteobacteria | 488/488 | 488 | *Vibrio* sp. KS30 |
| AB682172.1 | 0.0 | 99 | *Ferrimonas* sp. NBRC 104252 | Proteobacteria | 486/490 | 490 | *Ferrimonas* sp. KS31 |
| MH283837.1 | 0.0 | 100 | *Pseudovibrio denitrificans* strain SCSIO_43753 | Proteobacteria | 449/449 | 449 | *Pseudovibrio* sp. KS32 |
| MK308628.1 | 0.0 | 100 | *Vibrio coralliilyticus* strain SC53 | Proteobacteria | 500/500 | 500 | *Vibrio* sp. KSS33 |
| MK308628.1 | 0.0 | 99 | *Vibrio coralliilyticus* strain SC53 | Proteobacteria | 492/493 | 493 | *Vibrio* sp. KS34 |
| MH283839.1 | 0.0 | 100 | *Vibrio sinaloensis* strain SCSIO_43755 | Proteobacteria | 493/493 | 493 | *Vibrio* sp. KS35 |
| MH283839.1 | 0.0 | 100 | *Vibrio sinaloensis strain* SCSIO_43755 | Proteobacteria | 495/495 | 495 | *Vibrio* sp. KS36 |
| MH283839.1 | 0.0 | 99 | *Vibrio sinaloensis* strain SCSIO_43755 | Proteobacteria | 488/489 | 489 | *Vibrio* sp. KS37 |
| MH283839.1 | 0.0 | 99 | *Vibrio sinaloensis* strain SCSIO_43755 | Proteobacteria | 489/490 | 490 | *Vibrio* sp. KS38 |
| MH714891.1 | 0.0 | 100 | *Bacillus kochii* strain KB7 | Firmicutes | 482/482 | 482 | *Bacillus* sp. KS39 |
| MK308628.1 | 0.0 | 99 | *Vibrio coralliilyticus* strain SC53 | Proteobacteria | 481/482 | 482 | *Vibrio* sp. KS40 |
| KY655377.1 | 0.0 | 100 | *Spongiobacter* sp. strain EA276 | Proteobacteria | 487/487 | 487 | *Spongiobacter* sp. KS41 |
| MH283839.1 | 0.0 | 100 | *Vibrio sinaloensis* strain SCSIO_43755 | Proteobacteria | 480/480 | 480 | *Vibrio* sp. KS42 |
| MK308628.1 | 0.0 | 100 | *Vibrio coralliilyticus* strain SC53 | Proteobacteria | 480/480 | 480 | *Vibrio* sp. KS43 |
| MH283839.1 | 0.0 | 100 | *Vibrio sinaloensis strain* SCSIO_43755 | Proteobacteria | 495/495 | 495 | *Vibrio* sp. KS44 |
| MH283839.1 | 0.0 | 100 | *Vibrio* *sinaloensis* strain SCSIO_43755 | Proteobacteria | 495/495 | 495 | *Vibrio* sp. KS45 |
| MK346069.1 | 0.0 | 100 | *Bacillus* *pumilus* strain ASpB9 | Firmicutes | 460/460 | 460 | *Bacillus* sp. KS46 |
| MK611646.1 | 0.0 | 100 | *Bacillus toyonensis* strain B1 | Firmicutes | 488/488 | 488 | *Bacillus* sp. KS47 |
| MK156169.1 | 0.0 | 100 | *Bacillus mojavensis* strain DOK6 | Firmicutes | 473/473 | 473 | *Bacillus* sp. KS48 |
| MG719547.1 | 0.0 | 100 | *Bacillus aquimaris* strain L20 | Firmicutes | 482/482 | 482 | *Bacillus* sp. KS49 |
| MK156169.1 | 0.0 | 100 | *Bacillus mojavensis* strain DOK6 | Firmicutes | 482/482 | 482 | *Bacillus* sp. KS50 |
| MK106336.1 | 0.0 | 100 | *Bacillus megaterium* strain K13NAY002 | Firmicutes | 482/482 | 482 | *Bacillus* sp. KS51 |
| MK308628.1 | 0.0 | 99 | *Vibrio coralliilyticus* strain SC53 | Proteobacteria | 490/492 | 492 | *Vibrio* sp. KS52 |
| MH283839.1 | 0.0 | 100 | *Vibrio sinaloensis* strain SCSIO_43755 | Proteobacteria | 495/495 | 495 | *Vibrio* sp. KS53 |
| MH283839.1 | 0.0 | 100 | *Vibrio sinaloensis* strain SCSIO_43755 | Proteobacteria | 498/498 | 498 | *Vibrio* sp. KS54 |
| MK346068.1 | 0.0 | 100 | *Bacillus firmis* strain ASpB1 | Firmicutes | 487/487 | 487 | *Bacillus* sp. KS55 |
| MK346069.1 | 0.0 | 100 | *Bacillus pumilus* strain ASpB9 | Firmicutes | 482/482 | 482 | *Bacillus* sp. KS56 |
| MK346069.1 | 0.0 | 100 | *Bacillus pumilus* strain ASpB9 | Firmicutes | 462/462 | 462 | *Bacillus* sp. KS57 |
| AB682172.1 | 0.0 | 99 | *Ferrimonas* sp. NBRC 104252 | Proteobacteria | 485/489 | 489 | *Ferrimonas* sp. KS58 |
| MK318662.1 | 0.0 | 99 | *Vibrio harveyi* strain 2018B22 | Proteobacteria | 497/498 | 498 | *Vibrio* sp. KS59 |
| MK346069.1 | 0.0 | 99 | *Bacillus pumilus* strain ASpB9 | Firmicutes | 474/475 | 475 | *Bacillus* sp. KS60 |
| AB682172.1 | 0.0 | 99 | *Ferrimonas* sp. NBRC 104252 | Proteobacteria | 488/492 | 492 | *Ferrimonas* sp. KS61 |

KS: Kuwait sponge.

**Table S4.** Sampling site, date and taxonomic identification of sponge sample collected from Kuwait marine environment.

| Location | date | Number of samples | Sample ID | Taxonomy | | | |
| --- | --- | --- | --- | --- | --- | --- | --- |
|  |  |  |  | **Class** | **Order** | **Family** | **species** |
| Nuwaiseeb | 17^th^ Feb 2018 | 2 | 2NW,5NW | Demospongiae | Haplosclerida | Niphatidae | *Niphates* sp.1 |
| Kubbar Island | 1^st^ Aug 2017 | 1 | 2KI | Demospongiae | Haplosclerida | Niphatidae | *Niphates* sp.3 |
|  |  | 1 | 6KI | Demospongiae | Haplosclerida | Niphatidae | *Amphimedon* sp. |
|  |  | 2 | 1KI,5KI | Demospongiae | Haplosclerida | Chalinidae | *Haliclona* sp. |
|  |  | 1 | 4KI | Demospongiae | Chondrillida | Chondrillidae | *Chondrilla australiensis* |

**Table S5.** The documented numbers of sponges-associated prokaryotes raw 16S rRNA amplicon next generation sequencing reads, prokaryotic operational taxonomic units (OTUs) and genera.

| **Sponge species** | **Code** | **Mean read length bp** | **Number of reads bp** | **Total number of OTUs *** | **Total number of genera** |
| --- | --- | --- | --- | --- | --- |
| ***Haliclona* sp.** | 1KI** | 465 | 316,199 | 1,379 | 138 |
|  | 5KI | 466 | 235,203 | 1,266 | 123 |
| ***Niphates* sp.1** | 2NW*** | 470 | 192,875 | 1,917 | 215 |
|  | 5NW | 471 | 173,382 | 1,707 | 165 |
| ***Niphates* sp.3** | 2KI | 466 | 402,525 | 1,147 | 119 |
| ***Chondrilla australiensis*** | 4KI | 469 | 185,410 | 2,654 | 190 |
| ***Amphimedon* sp.1** | 6KI | 436 | 74,801 | 3,708 | 440 |

*****excluding singletons and doubletons.

** KI= Kubbar Island (August, 2017).

***NW= Nuwaiseeb intertidal (February, 2018).

**Table S6.** Number of spots containing the filtered bacterial sequence (cultured bacterial isolates) found in *Haliclona* sp. NGS reads (SRX14840950).

| **Bacterial genera** | **NCBI accession number** | **Number of spots containing the filtered sequence** |
| --- | --- | --- |
| *Shewanella* sp*.* KS1 | MK558635 | 17 |
| *Shewanella* sp*.* KS16 | MK558650 | 17 |
| *Shewanella* sp*.* KS18 | MK558652 | 17 |
| *Vibrio* sp. KS2 | MK558636 | 1371 |
| *Vibrio* sp. KS3 | MK558637 | 1359 |
| *Vibrio* sp. KS4 | MK558638 | 1 |
| *Vibrio* sp. KS7 | MK558641 | 1371 |
| *Vibrio* sp. KS9 | MK558643 | 930 |
| *Vibrio* sp. KS10 | MK558644 | 1371 |
| *Vibrio* sp. KS11 | MK558645 | 1361 |
| *Vibrio* sp. KS14 | MK558648 | 64 |
| *Vibrio* sp. KS17 | MK558651 | 1 |
| *Vibrio* sp. KS19 | MK558653 | 7 |
| *Vibrio* sp. KS22 | MK558656 | 1371 |
| *Vibrio* sp. KS23 | MK558657 | 1346 |
| *Vibrio* sp. KS25 | MK558659 | 1365 |
| *Vibrio* sp. KS26 | MK558660 | 50 |
| *Vibrio* sp. KS27 | MK558661 | 931 |
| *Vibrio* sp. KS28 | MK558662 | 931 |
| *Vibrio* sp. KS29 | MK558663 | 1366 |
| *Vibrio* sp. KS30 | MK558664 | 930 |
| *Vibrio* sp. KS33 | MK558667 | 1365 |
| *Vibrio* sp. KS34 | MK558668 | 1371 |
| *Vibrio* sp. KS35 | MK558669 | 1371 |
| *Vibrio* sp. KS36 | MK558670 | 1371 |
| *Vibrio* sp. KS37 | MK558671 | 1 |
| *Vibrio* sp. KS38 | MK558672 | 1371 |
| *Vibrio* sp. KS40 | MK558674 | 1365 |
| *Vibrio* sp. KS42 | MK558676 | 1361 |
| *Vibrio* sp. KS43 | MK558677 | 1361 |
| *Vibrio* sp. KS44 | MK558678 | 1361 |
| *Vibrio* sp. KS45 | MK558679 | 1361 |
| *Vibrio* sp. KS52 | MK558686 | 22 |
| *Vibrio* sp. KS53 | MK558687 | 1360 |
| *Vibrio* sp. KS54 | MK558688 | 1365 |
| *Vibrio* sp. KS59 | MK558693 | 11 |
| *Pseudovibrio* sp. KS8 | MK558642 | 1 |
| *Pseudovibrio* sp. KS21 | MK558655 | 1 |
| *Pseudovibrio* sp. KS32 | MK558666 | 1 |
| *Ferrimonas* sp. KS5 | MK558639 | 1651 |
| *Ferrimonas* sp. KS6 | MK558640 | 1728 |
| *Ferrimonas* sp. KS15 | MK558649 | 2 |
| *Ferrimonas* sp. KS20 | MK558654 | 1721 |
| *Spongiobacter* sp. KS12 | MK558646 | 89 |
| *Spongiobacter* sp. KS13 | MK558647 | 1 |
| *Spongiobacter* sp. KS41 | MK558675 | 1 |
| *Bacillus* sp. KS39 | MK558673 | 13 |
| *Bacillus* sp. KS46 | MK558680 | 23 |
| *Bacillus* sp. KS47 | MK558681 | 12 |
| *Bacillus* sp. KS48 | MK558682 | 1 |
| *Bacillus* sp. KS49 | MK558683 | 13 |
| *Bacillus* sp. KS50 | MK558684 | 23 |
| *Bacillus* sp. KS51 | MK558685 | 12 |
| *Bacillus* sp. KS55 | MK558689 | 26 |
| *Bacillus* sp. KS56 | MK558690 | 23 |
| *Bacillus* sp. KS57 | MK558691 | 23 |
| *Bacillus* sp. KS60 | MK558694 | 23 |

**Table S7.** Antimicrobial activity of bacterial isolates obtained from *Haliclona* sp. 1Kl.

| **Isolate name /code** | **Zone of inhibition against tested microorganisms (mm)** | | |
| --- | --- | --- | --- |
|  | ***Staphylococcus aureus*** | ***Bacillus subtilis*** | ***Candida albicans*** |
|  | **Mean(minimum-maximum) standard deviation** | | |
| *Bacillus* sp. KS5d2 | 10(10-12)1 | 9(9-10)0.6 | - |
| *Bacillus* sp. KS5b1 | 10(10-11)0.05 | 1(1-1.1)0.5 | 11(10-13)1.1 |
| *Bacillus* sp. KS50 | 9(9-10)0.6 | 8(8-9)0.6 | - |
| *Bacillus* sp. KS47 | - | - | 10(10-11)0.5 |
| *Bacillus* sp. KS57 | 9(8-10)1 | 8(8-9)0.6 | - |
| *Bacillus* sp. KS46 | 11(11-12)0.6 | - | - |
| *Bacillus* sp. KS56 | - | 11(11-12)0.6 | - |
| Ampicillin | 20 | 15 |  |
| Kanamycin | 26 | 30 |  |
| Penicillin-G | 21 | 18 |  |
| Cycloheximide |  |  | 55 |

- **No microbial activity, ≥10 mm moderate activity, ≤10 mm weak activity.**

**References:**

Esteves, A.I., Hardoim, C.C., Xavier, J.R., Gonçalves, J.M., and Costa, R. (2013). Molecular richness and biotechnological potential of bacteria cultured from Irciniidae sponges in the north-east Atlantic. FEMS Microbiology Ecology, 85(3): 519-536. doi: 10.1111/1574-6941.12140

Knobloch, S., Johannsson, R., and Marteinsson, V. (2019). Co-cultivation of the marine sponge *Halichondria panicea* and its associated microorganisms. *Scientific reports*, *9*(1): 1-11. Accessed at <https://www.nature.com/articles/s41598-019-46904-3>

Laport, M. S., Bauwens, M., de Oliveira Nunes, S., Willenz, P., George, I., and Muricy, G. (2017). Culturable bacterial communities associated to Brazilian *Oscarella* species (Porifera: Homoscleromorpha) and their antagonistic interactions. *Antonie Van Leeuwenhoek*, *110*(4): 489-499. doi: 10.1007/s10482-016-0818-y

Matobole, R. M., Van Zyl, L. J., Parker‐Nance, S., Davies‐Coleman, M. T., and Trindade, M. (2017). Antibacterial activities of bacteria isolated from the marine sponges *Isodictya compressa* and *Higginsia bidentifer*a collected from Algoa Bay, South Africa. Marine drugs, 15(2): 47. doi:10.3390/md15020047

Öner, Ö., Ekiz, G., Hameş, E., Demir, V., Gübe, Ö., Özkaya, F., Yokes, M., Uzel, A., and Bedir, E. (2014). Cultivable sponge- associated actinobacteria from coastal area of eastern mediterranean sea. *Advances in Microbiology*, 4: 306–316. doi: 10.4236/ aim.2014.46037

Rajasabapathy, R., Ghadi, S. C., Manikandan, B., Mohandass, C., Surendran, A., Dastager, S. G., Meena, R., and James, A. (2020). Antimicrobial profiling of coral reef and sponge-associated bacteria from southeast coast of India. Microbial Pathogenesis, 141: 103972. doi: 10.1016/j.micpath.2020.103972

Selvin, J., Gandhimathi, R., Kiran, G. S., Priya, S. S., Ravji, T. R., and Hema, T. A. (2009). Culturable heterotrophic bacteria from the marine sponge *Dendrilla nigra*: isolation and phylogenetic diversity of actinobacteria. Helgoland Marine Research, 63(3): 239-247. doi: 10.1007/s10152-009-0153-z

Steinert, G., Whitfield, S., Taylor, M. W., Thoms, C., and Schupp, P. J. (2014). Application of diffusion growth chambers for the cultivation of marine sponge-associated bacteria. *Marine Biotechnology*, *16*(5), 594-603. doi: 10.1007/s10126-014-9575-y
